# Truly‐Biocompatible Gold Catalysis Enables Vivo‐Orthogonal Intra‐CNS Release of Anxiolytics

**DOI:** 10.1002/anie.202111461

**Published:** 2021-11-18

**Authors:** M. Carmen Ortega‐Liebana, Nicola J. Porter, Catherine Adam, Teresa Valero, Lloyd Hamilton, Dirk Sieger, Catherina G. Becker, Asier Unciti‐Broceta

**Affiliations:** ^1^ Cancer Research UK Edinburgh Centre Institute of Genetics & Cancer University of Edinburgh Edinburgh EH4 2XU UK; ^2^ Centre for Discovery Brain Sciences The Chancellor's Building University of Edinburgh Edinburgh EH16 4SB UK; ^3^ Center for Regenerative Therapies Technische Universität Dresden 01307 Dresden Germany

**Keywords:** behavioral activity, bioorthogonal, catalysis, gold, prodrug

## Abstract

Being recognized as the best‐tolerated of all metals, the catalytic potential of gold (Au) has thus far been hindered by the ubiquitous presence of thiols in organisms. Herein we report the development of a truly‐catalytic Au‐polymer composite by assembling ultrasmall Au‐nanoparticles at the protein‐repelling outer layer of a co‐polymer scaffold via electrostatic loading. Illustrating the in vivo‐compatibility of the novel catalysts, we show their capacity to uncage the anxiolytic agent fluoxetine at the central nervous system (CNS) of developing zebrafish, influencing their swim pattern. This bioorthogonal strategy has enabled ‐for the first time‐ modification of cognitive activity by releasing a neuroactive agent directly in the brain of an animal.

## Graphical abstract

Making drugs in the brain! Fully‐biocompatible Au catalysis enables for the first time the localized uncaging of a neuroactive agent, the anxiolytic drug fluoxetine, directly in the head of zebrafish by using catalytically‐enhanced Au‐polymer composites as safe catalytic reactors.

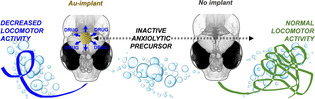

Bioorthogonal chemistries have rapidly expanded the repertoire of methods that researchers can access to interrogate and modulate cell biology.^[1]^ Within this ever‐growing field, nonbiological transition metals emerged as catalytic tools to transform endo‐ or exogenous molecules in living cells and organisms without the need to rely on natural enzymes.^[2]^ Among the experimental uses of bioorthogonal catalysts, the abiotic activation of drug precursors (triggered by cleavage or bond‐forming reactions)[^1c^, ^3^] has demonstrated its preclinical applicability, for example, to kill cancer cells and tumors^[4]^ or to reduce local inflammation.^[5]^ Nonetheless, the potential of such a therapeutic modality to treat disorders within the complex biochemical environment of the most sensitive organs, such as the brain, remains yet untested.

Among the transition metal catalysts that have proved their functional compatibility with biological conditions (e.g. Ru, Pd, Au, Cu, Fe),^[6]^ metallic Au stands out because of its excellent tolerability and alkynophile properties, which makes it potentially suitable for non‐oncological prodrug activation strategies. However, its avidity to form quasi‐covalent bonds with biogenic thiols hinders its use as a bioorthogonal catalyst in free form. Previous research has shown that Au nanoparticles (Au‐NPs) generated within polymer supports can mediate depropargylation reactions in cell culture.^[2o]^ Despite this encouraging finding, the chemical properties of current Au‐NP‐loaded devices rapidly undergo deactivation in serum‐containing media,[^2o^, ^7^] which deter them from being used to release drugs in vivo. One reason for this effect is that only those Au‐NPs that are closer to the surface of the solid support can effectively contribute to the reactivity of the overall heterogeneous system and this relatively‐small fraction of Au‐NPs is exposed to and swiftly poisoned by thiol‐rich proteins present in the surrounding medium. In fact, without photothermic stimulation,^[8]^ the use of metallic Au as a true catalyst in living systems is yet to be demonstrated.

Driven by the challenge of developing truly‐biocompatible catalysts able to synthesize drugs in the most complex and fragile in vivo settings, we set to investigate how to enhance the catalytic properties of Au‐NP‐based catalysts. Rather than generating Au‐NPs in situ within a polymeric framework (method used in previous studies[^2o^, ^7^]), a process that reduces control of particle size and distribution, we rationalized that catalytic performance and NP loading could be maximized by doping a solid support with preformed Au‐NPs of well‐defined dimensions. Thus, we first investigated the optimal particle size of Au‐NPs that favors depropargylation reactions in aqueous media. The catalytic properties of a variety of Au‐NPs (Table S1, and Figure S1, Supp. Inf.) were tested using the off‐on fluorescent probe Pro‐Res,^[2s]^ which upon *O*‐propargyl cleavage releases strongly fluorescent resorufin. Au‐NPs of 2.9 nm in average diameter, synthesized by treatment of HAuCl_4_ (Au source) and tetrakis(hydroxymethyl) phosphonium chloride (precursor of the reductive and stabilizing agent) in a NaOH aqueous solution (Figure 1 a), demonstrated superior catalytic properties than larger Au‐NPs in PBS at 37 °C (Figure S1, Supp. Inf.). As shown in Figure S2 (Supp. Inf.), these ultrasmall negatively‐charged metallic particles possess a highly‐crystalline structure and low polydispersity index. Even if their catalytic properties in saline buffer solution are excellent (>99 % yield, 24 h), their reactivity is significantly reduced in the presence of serum (64 % yield, 24 h), which is in agreement with the anticipated blockade of the environment‐exposed catalytic sites of the metal by reaction with thiol‐containing biomolecules.

**Figure 1:**
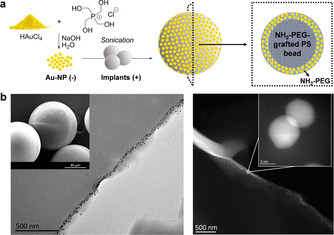
Preparation and characterization of Au‐microimplants. a) Synthesis of Au‐NPs and Au‐microimplants. b) SEM/TEM (left) and HAADF‐STEM (right) images of ultramicrotome cross‐sections of Au‐microimplants at different magnifications.

To create efficient microreactors that enable spatial control of dye/drug release, freshly‐prepared negatively‐charged Au‐NPs were loaded at the “crust” of positively‐charged NH_2_‐PEG‐grafted PS microimplants (75 μm in average diameter) by treating them in water under sonication (Figure 1 a, see full protocol in the Supp. Inf.). This electrostatic loading strategy aimed to accumulate Au‐NPs at the positively‐charged outer amphiphilic layer of the microimplant, to trap and shield the NPs with a protein repelling material and, at the same time, facilitate small molecule access to the active sites of the metal‐polymer composite. Characterization was carried out by electron microscopy and energy‐dispersive X‐ray micro‐analysis to study NP distribution (Figure 1 b) and elemental composition (Figure S3, Supporting Information), respectively. Images noticeably show NPs trapped near the surface of the solid support. Au content was determined to be 1.9 % (w/w), >10‐fold higher than that obtained with previous protocols.[^2o^, ^7^]

Functional characterization of the new devices was performed by fluorogenic monitoring of green light‐emitting fluorophore **1** ^[10]^ upon Au‐mediated cleavage of the novel off‐on prodye **2** (Figure 2 a). Because many neuroactive agents (including fluoxetine, **3**) feature an NH group in their structure that is essential to interact with their target,^[11]^ we designed a Au‐labile nitrobenzodioxazole (NBD)‐based profluorophore by carbamate‐masking the secondary amine of **1**. Figure S4 shows that **2** emits negligible fluorescence under 480 nm excitation compared to **1** (>300‐fold difference at 536 nm), a consequence of decreasing the electron donating character of the 4‐ethylamino moiety of **1**. Reactions were performed by incubating Au‐microimplants with **2** at 37 °C with/without serum (Figure 2 a). As shown in Figure 2 b, contrary to free‐form Au‐NPs, the reactivity of the microimplants is accelerated in the presence of serum, achieving >40 % yield in 4 h and full reaction completion in 24 h using sub‐stoichiometric quantities of Au (TON≥5). To our delight, recovery of the devices and re‐use under the same reaction conditions confirmed the catalytic capabilities of the devices (Figure 2 c and Figure S5, Supporting Information), with recycling being repeated eight times with minimal loss of reactivity (overall TON≥40). This catalytic enhancement, consequence of the optimal particle size and new loading strategy, is remarkable as it overcomes the operability limitations of prior Au‐based catalysts and proves the capacity of Au to mediate catalytic depropargylations under biocompatible conditions within a suitable nanoenvironment. Exposure of Au‐microimplants to a range of biomolecules (Figure S6,7, Supporting Information), including glucose, ascorbate and thiol‐containing reagents (glutathione and cysteine), shows that the catalytic properties of the devices are only affected by thiols at concentrations above plasma levels.^[2o]^

**Figure 2:**
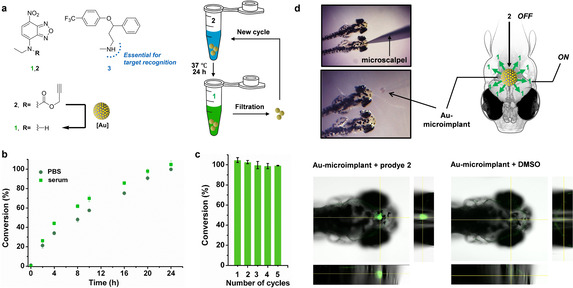
a) Au‐triggered conversion of Poc‐masked prodye **2** into dye **1** and structure of **3**. b) Fluorogenic reaction of **2** (50 μM) and Au‐microimplants (0.1 mg mL^−1^=9.6 μM in Au content) in PBS:methanol (70:30) with or without serum (pH 7.4, 37 °C). Conversion (%) was measured at different time points from fluorescence intensity measurements (*λ*_ex/em_=485/535 nm) and calculated using a standard curve of **1**. c) Recycling test. Au‐microimplants (0.1 mg mL^−1^) were recovered after each reaction cycle and re‐used under the reaction conditions described before. Conversion (%) was measured at 24 h. d) Intracranial implantation of an Au‐microimplant in zebrafish and confocal analysis of the CNS generation of green fluorescence in the presence (left) or absence (right) of pro‐dye **2**.

Encouraged by these results, we embarked on a series of experiments towards the goal of releasing a psychoactive drug at the CNS of zebrafish, an animal model used to test the effects of neuro‐therapeutics via monitoring swimming behavior.^[9]^ By enabling the bioorthogonal modulation of a superior organism at the cognitive (supra‐cellular) level, rather than at the cellular level widely attempted by us and others,[^1^, ^2^, ^3^, ^4^, ^5^] we sought to challenge the full potential of our catalytically‐enhanced biocompatible catalyst. First, to assess potential harms caused by the Au‐microimplants, their biocompatibility was tested in cell lines, including SH‐SY5Y cells (model of CNS origin). Cell viability assays showed no signs of toxicity at any of the concentrations tested (Figure S8, Supporting Information). Surgical insertion of a single Au‐microimplant in the brain of 3‐dpf zebrafish confirmed that the size of the Au‐implants were compatible for cranial implantation (Figure 2 d). To determine if the presence of the implant alters fish behavior, zebrafish movements were tracked and analyzed in real time after surgery recovery (Figure S9, Supporting Information). Evaluation of swimming distance and speed confirmed that the presence of the implant did not modify zebrafish activity. No signs of toxicity were observed in any of the fish (*n*=20). To validate the catalytic properties of the devices in vivo, prodye **2** was incubated with embryos grafted with an Au‐microimplant. As shown in Figure 2 d, confocal microscopy confirmed the presence of strong fluorescent signal only at the site of the Au‐microimplant, confirming the intra‐CNS generation of dye **1**. In contrast, no fluorescence was observed in implant‐grafted zebrafish in the absence of **2**.

Next, we selected and tested neuroactive agents known to influence zebrafish cognitive activity: NMDA, GABA and fluoxetine.^[9]^ Among these drugs (see Figure S10, Supporting Information), the selective serotonin reuptake inhibitor fluoxetine, **3** (a.k.a. Prozac), produced a highly significant reduction of zebrafish locomotor activity (see Movie S1). Since **3** contains a secondary NH group essential for its bioactivity,^[11]^ we synthesized an Au‐labile carbamate‐protected derivative by reacting propargyloxycarbonyl chloride and **3** in the presence of triethylamine, to give rise prodrug **4** in good yield (91 %). Study of the bioactivity of prodrug **4** compared with parent drug **3** in 5‐HT2B overexpressing cells determined that, whereas **3** displays agonist activity, prodrug **4** is completely inactive, even at the top dose of 100 μM (Figure S11a–c, Supporting Information). Zebrafish studies confirmed that **4** does not elicit any effect on zebrafish locomotor activity (Figure S11d,e, Supporting Information). The capacity of the Au‐implants to convert **4** into **3** in vitro was confirmed by LCMS (Figure S12,13, Supporting Information).

Finally, the in vivo applicability of heterogeneous Au catalysis was validated by testing the capacity of an Au‐microimplant to inhibit zebrafish locomotor activity by the intracranial release of the anxiolytic drug **3** (Figure 3 a). Microimplants were carefully grafted (one bead per fish) into the head of 3‐dpf larvae. Prodrug **4** was added to the medium and zebrafish movements monitored after treatment (*n*=20). Zebrafish treated only with **4** (no implant) and Au‐microimplant grafted larvae without prodrug were used as negative controls. Zebrafish treated with **3** were used as positive controls. As shown in Figure 3 b,c, negative controls did not alter zebrafish behavior relative to the untreated control. In line with the effect observed by direct treatment with **3**, the locomotor activity of zebrafish grafted with Au‐microimplants and treated with **4** decreased significantly relative to the untreated controls (Figure 3 c), evidence of the local generation of fluoxetine **3**. Additional control studies using zebrafish implanted with naked beads and incubated with prodrug **4** further demonstrated that Au is essential to release **3** and modify animal behavior (Figure S14, Supporting Information).

**Figure 3:**
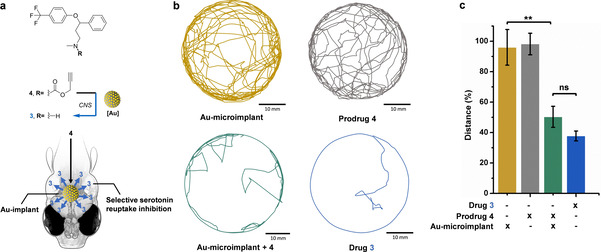
a) Bioorthogonal intra‐CNS control of zebrafish locomotor activity by localized Au‐mediated generation of anxiolytic **3** from inactive precursor **4** in the head of zebrafish. b) Representative images of the tracked distances of individual zebrafish in a cell culture dish (35 mm×10 mm) under different treatments. [Prodrug/drug]=50 μM. Zebrafish movements were analyzed using the EthoVision XT 8.5 software (Noldus). c) % distance swum by zebrafish embryos under treatment relative to the DMSO‐treated control. Error bars: ±SEM, *n*=20; ** *P*<0.01.

In conclusion, we have developed a novel Au‐based heterogeneous catalyst that is fully compatible with living systems, both in terms of safety and functionality. To achieve this, a novel loading strategy was implemented to increase the abundance of active metal centers at the outer layer of a polymer scaffold. The properties of the Au‐microimplants were tested in vitro and in vivo with a new Au‐activatable fluorescence precursor. Of note, the small and readily‐amenable chemical structure^[10b]^ of this NBD‐based prodye enables the design of a palette of probes featuring fluorescence emission from green to far red, a useful characteristic to perform multiplexed screenings or in vivo studies in larger animal models. Finally, we have shown that the swimming behavior of zebrafish embryos can be modulated by the bioorthogonal release of an anxiolytic agent directly in their head, something never achieved before with bioorthogonal tools. This proof‐of‐concept study demonstrates that Au catalysts can mediate abiotic reactions at neural networks without causing harm or interfering with the complex biochemistry of this sensitive environment, making it a true *vivo*‐compatible catalyst. The present work expands the scope of Au chemistry and *vivo*‐orthogonal catalysis, offering a new methodology to study neurological function by producing bioactive agents exclusively at the brain of zebrafish (thus avoiding the noise produced at the peripheral nervous system). Given the possibility to translate this strategy to peripheral damaged nerves (outside the brain), this research opens up a future route to treat localized neurological disorders, such as neuropathic pain.

## Conflict of interest

The authors declare no conflict of interest.

## Supporting information

As a service to our authors and readers, this journal provides supporting information supplied by the authors. Such materials are peer reviewed and may be re‐organized for online delivery, but are not copy‐edited or typeset. Technical support issues arising from supporting information (other than missing files) should be addressed to the authors.

Supporting Information

Supporting Information
