# Supplementary material for: Truly‐Biocompatible Gold Catalysis Enables Vivo‐Orthogonal Intra‐CNS Release of Anxiolytics
Source: Angew Chem Int Ed Engl. 2021 Nov 18;61(1):e202111461. doi: 10.1002/anie.202111461 (PMC9299494; doi:10.1002/anie.202111461)
Supplement: Supplementary file 1 — Supporting Information [file ANIE-61-0-s001.pdf]

Angewandte Chemie
Eine Zeitschrift der Gesellschaft Deutscher Chemiker

Supporting Information

Truly-Biocompatible Gold Catalysis Enables Vivo-Orthogonal Intra-CNS Release of Anxiolytics

*M. Carmen Ortega-Liebana[+], Nicola J. Porter[+], Catherine Adam, Teresa Valero, Lloyd Hamilton, Dirk Sieger, Catherina G. Becker, and Asier Unciti-Broceta**

anie_202111461_sm_miscellaneous_information.pdf
anie_202111461_sm_Movie_S1.mov

TABLE OF CONTENTS

1. **General Information** ……………………………………………………………………………2

2. **Experimental Procedures and characterizations** ……………………………………...3

3. **Biological studies** ………………………………………………………………………...10

4. ***In vivo* experiments** ……………………………………..…………………………….…..11

5. **Supplementary References** ……………………...………………….………………… 13

6. **Supplementary Tables and Figures** …………………………………………………. 14

1. General Information

Materials. Chemical and solvents were purchased from Fisher Scientific, Sigma-Aldrich or VWR International Ltd. TentaGel® HL, NH_2 (HL12902) and OH (HL12903), resins were purchased from Rapp Polymere GmbH. Resorufin (95% purity) and fluoxetine hydrochloride **1** (98% purity) are commercially available and were purchased from Fluorochem UK. D-Glucose anhydrous (99% purity) and L-Ascorbic acid (99% purity) were purchased from Fisher Scientific and Scientific Laboratory Supplies, repectively. 4-Chloro-7-nitrobenzofurazan (NBD-Cl, 98% purity) was purchased from Alfa Aesar. GABA (γ-Aminobutyric acid, ≥99% purity), NMDA (>98% purity), L-cysteine (97% purity) and L-glutathione reduced (>98% purity) were purchased from Sigma-Aldrich and Cayman Chemical, respectively. Pro-Resorufin (**Pro-Res**) was synthesized as previously reported,[1] with slight modifications to the purification method.

Characterisation. NMR spectra were recorded at 300 K on a 500 MHz Bruker Avance III HD spectrometer. Chemical shifts are reported in parts per million (ppm) relative to the solvent peak (^{1}H NMR DMSO-d_6 δ2.50 ppm; ^{13}C-NMR DMSO-d_6 δ39.52 ppm). Data are presented as follows: chemical shift (ppm), multiplicity (s = singlet, d = doublet, t = triplet, m = multiplet, q= quartet), coupling constant *J*, and integration. R_f values were determined on Merck TLC Silica gel 60 F_{254} plates under a 254 nm UV source. Purifications were carried out by flash column chromatography using commercially available Biotage® Sfär silica column (60 μm particle size - 10 g). High-Resolution Mass Spectrometry was performed with a Bruker MicrOTOF II. The optical and fluorescent properties of NBD-NHEt (**1**) and **POC-NBD (2)** were analyzed by a NanoDrop™ 2000c Spectrophotometer and a NanoDrop™ 3300 Fluorospectrometer, respectively, both acquired from Thermo Scientific™. The purity of **POC-NBD** (used in cell and zebrafish studies) was >99.9%, as measured by HPLC using an Agilent 1260 Infinity II Preparative LC/MSD system coupled to an Evaporative Light Scattering detector (ELSD). HPLC method: eluent A: water and formic acid (0.1%); eluent B: methanol and formic acid (0.1%); and A/B = 95:5 to 5:95 in 4 min and isocratic 2 min (flow = 1 mL/min). The purity of prodrug **4** (used in cell and zebrafish studies) was >99%, as measured by HPLC using an Agilent 1260 Infinity II Preparative LC/MSD system coupled to an Evaporative Light Scattering detector (ELSD). HPLC method: eluent A: water and formic acid (0.1%); eluent B: acetonitrile and formic acid (0.1%); and A/B = 95:5 to 5:95 in 4 min and isocratic 2 min (flow = 1 mL/min). Prodrug-into-drug conversion experiments were conducted using the same HPLC equipment and method described above. Stock solutions (150 mM) were prepared in DMSO. The optical properties of **Au-NPs** were analyzed by a NanoDrop™ 2000c spectrophotometer (Thermo Scientific™), and **Au** loading of the implants was determined by ICP-OES (Perkin Elmer 8300 DV). The hydrodynamic diameter of the nanoparticles was determined by DLS using a Malvern Zetasizer Nano-S (Malvern). The Zeta potential (ζ potential) of the nanoparticles was performed on a Zetasizer Nano-ZS (Malvern). SEM and TEM images were obtained using a FEI Inspect F50 microscope equipped with an EDX analytical system, and a Titan (Thermofisher Scientic, formely FEI) with a Field Emission Gun operating at 300 kV, respectively.

2. Experimental Procedures and Characterizations

Synthesis of Au-NPs and testing of catalytic properties. The size, capping agents, and crystalline structure of metallic nanoparticles are factors that can affect their catalytic properties. In a preliminary screening, we synthesized monodisperse quasi-spherical particles in a size range of 15 to 150 nm in diameter by varying the Au:citrate ratio in the reaction parameters.[2] As shown in **Table S1**, smaller citrate-stabilized particles showed higher catalytic activity by fluorogenic studies compared to larger sizes. Therefore, having established that smaller size Au-NPs have superior catalytic activity, we tested a number of synthetic protocols to control NP growth and morphology by changing the reducing and capping agents (see **Table S2**). Monodisperse Au-NPs with different characteristics were prepared and analyzed by DLS, UV-vis, ζ potential and HAADF-STEM.

Synthesis of citrate-based **NPs**: **Au-NP-1** and **Au-NP-2** were prepared as previously described[2-3], respectively, without modifications.

Synthesis of negatively charged **Au-NP-3 (a.k.a. Au-NPs in the main manuscript)**: NPs were prepared as previously described[4] with slight modifications. 50 mL glass vial was cleaned with aqua regia to dissolve and lift any traces of metal deposits off the glass surfaces. Distilled water (45 mL) was added and stirred vigorously with a cylindrical magnetic stir bar with pivot ring for the addition of aqueous sodium hydroxide (0.2 M, 1.5 mL) followed by tetrakis(hydroxymethyl)phosphonium chloride (THPC, 80 wt%) (85 mM, 1 mL). After 5 min stirring, freshly dissolved $HAuCl_4 \cdot 3H_2O$ (≥99.9% trace metals basis) (25 mM, 2 mL) was added and the colour changed from yellow to dark brown within seconds. The reaction mixture was stirred overnight at ambient temperature to allow for complete formation of gold cores, protecting the mixture from light to prevent the photothermal decomposition of the precursors.[5] In the particle nucleation mechanism, the *in situ* generation of formaldehyde from THPC under basic conditions favours the reduction of Au (III) to Au (0). The formaldehyde acts as an active reducing agent, oxidizing to formic, and generating and stabilizing **Au-NPs** with negative charge (average ζ potential value is – 19.9 ± 4.2 mV), as previously reported.[4, 6] Full characterization of **Au-NP-3** is shown in **Figure S2**. Synthesis of HTPC-based **Au-NPs** from **Table S1**: Au-NPs were prepared as described above for **Au-NP-3**, maintaining the concentration of the Au (III) salt precursor and the addition volumes of all the reagents. To obtain different particle sizes, the concentration of the reducing agent and NaOH (THPC-NaOH) were modified as follows: 76.5mM:0.17M, 68mM:0.13M, 64mM:0.1M, resulting in Au-NP with sizes of 9.6, 19.7 and 25.4 nm, respectively. Of note, the larger NPs (19.7 and 25.4 nm) were stable for no more than 24 h. Therefore, after synthesis, the NPs were centrifuged, resuspended in water, and tested immediately with the corresponding pro-dye.

The catalytic properties of **Au-NP-1**, **Au-NP-2** and **Au-NP-3** described above were tested using the off/on fluorescent probe **Pro-Res**, which upon *O*-propargyl cleavage releases strongly fluorescent resorufin. For this screening, a concentration of nanoparticles of 40 µg/mL was added to an aqueous solution of **Pro-Res** at 40 µM. Either PBS or PBS + 10% of serum (FBS) were used as reaction media. Reactions were shaken at 700 rpm and 37 ºC in a Thermomixer and fluorescence intensity analyzed after 24 h in a PerkinElmer EnVision 2101 multilabel reader (Ex/Em: 550 /580 nm). Samples were repeated in triplicate. The results are shown in **Figure S1**. Crystalline **Au-NP-3** featuring decahedral structures demonstrated superior catalytic properties in comparison with larger nanoparticles, synthesized and stabilized by SC (**Au-NP-1**), and the small faceted Au NPs (twinned nature), synthesized and stabilized by SC and TA (**Au-NP-2**).

Based on these the fluorogenic studies, **Au-NP-3** (**Au-NPs**) (simply named as **Au-NPs** from now on) were selected for preparing the implants.

Synthesis and characterization of Au-implants. Solid-supported gold catalysts were prepared by deposition of ultra-small **Au-NPs** on the surface of a PEG-grafted low-cross-linked polystyrene matrix. Briefly, 10 mg of TentaGel® HL amino-functionalized resins (75 microns) was dispersed in 1.5 mL of the freshly prepared **Au-NPs** suspension. After sonicating the mixture for 10 min, the resulting resins were collected by centrifugation (5 min at 8000 rpm). This procedure was repeated for a second time and the suspension was stirred at ambient temperature for 12 h in the dark using an eppendorf IKA rotary shaker loopster digital. Subsequently, the **Au-implants** were collected by centrifugation (5 min at 8000 rpm), washed several times with Milli-Q water, and re-dispersed in water until further use.

Electron microscopy analyses. **Au-microimplants** were first infiltrated and embedded in a EMBed812 resin polymerizing at 60ºC for 24 h. Then, before curing the resin block, it was stained with OsO_4 (4 wt.% in H_2O) for 1 h to harden the **Au-microimplants** and ease sectioning. Then, semithin and ultrathin sections of the **Au-microimplant** were obtained with a diamond knife (ultra 35°, Diatome) using an Ultramicrotome (Leica EM UC7). For characterization by SEM, the semithin sections (500-1000 nm in thickness) were placed on a pin stub with carbon tape and coated with 15 nm of carbon. Previously, stubs were glow discharged (30s, 15 mA) to enable the sections to be mounted as flat as possible. **Au-microimplant** blocks after sectioning were also mounted on stubs and coated with 15 nm of carbon to be observed by SEM, using a FEI Inspect F50 microscope and 10-20kV of acceleration voltage. This microscope is equipped with different SEM detectors that obtain: 1) SEM images and composition by using a Back Scattering Electron Detector and 2) elemental chemical analysis by energy-dispersive X-ray microanalysis (EDS). The ultrathin sections (50-70 nm in thickness) were placed on a carbon film on copper grid (150 mesh) and allowed to dry in air. TEM observation was conducted using a Titan (Thermofisher) with a Field Emission Gun operating at 300 kV at the LMA-INA-University of Zaragoza facilities with the assistance of Dr Fernandez-Pacheco.

The microscope spherical aberration corrector (CESCOR-CEOS) allows a point resolution of 0.8 Å. The microscope is fitted with a High-Angle Annular Dark Field (HAADF) detector (Fischione) to operate in Scanning Transmission Electron Microscopy (STEM) mode with Z-contrast imaging.

Quantification of Au content by inductively coupled plasma optical emission spectrometry (ICP-OES). Au-microimplants of different batches (1 mg of sample) were digested with 1 mL of freshly-prepared aqua regia and the samples were left for digestion for 30 min. Afterwards, 50 µL of each sample plus 50 µL of aqua regia was diluted to 4 mL with Mili-Q water to achieve a final concentration of acid of 2.5 % *v/v*. Au quantification was done on a Perkin Elmer 8300 DV. Calibrations were performed employing Au standards in the same background solution (2.5 % aqua regia) with excellent correlations. Samples were measured in triplicate.

Sample	ICP-OES Au 242.797 (mg/mL)	% rsd	mg Au/mg Resin	Au % *wt*/*wt*
**Au-**microimplant **batch 1**	0.210	0.5%	0.0168	1.68
**Au-**microimplant **batch 2**	0.233	0.4%	0.0187	1.87
**Au-**microimplant **batch 3**	0.249	1.3%	0.0199	1.99
**Au-**microimplant **batch 4**	0.222	0.5%	0.0178	1.78
**Au-**microimplant **batch 5**	0.237	0.2%	0.0190	1.90
**Au-**microimplant **batch 6**	0.265	0.7%	0.0212	2.12
**Mean**			0.0188	**1.9 % Au**
Blank	0.000			
0.5 ppm Check	0.486	2.1%		
0.1 ppm Check	0.104	0.3%		

Synthesis and characterization of Pro-Res

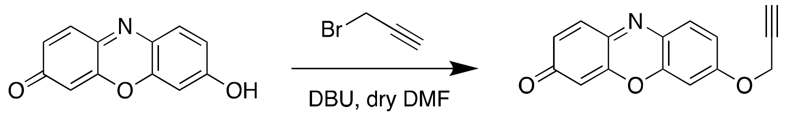

Pro-Res was synthesized as previously reported,[1] with slight modifications to the purification method. The crude was purified by silica gel column chromatography MeOH (2.5% → 5% v/v)-CH_2Cl_2 instead of via semipreparative TLC chromatography to give the desired product (7-Propargyloxy-3H-phenoxazin-3-one) as an orange solid (48 mg, 46% yield). **R*f*** = 0.35 (5% MeOH in CH_2Cl_2). **^{1}H NMR** (500 MHz, DMSO-d$_6$) δ = 7.82 (d, *J* = 8.9 Hz, 1H), 7.55 (d, *J* = 9.9 Hz, 1H), 7.18 (d, *J* =2.7 Hz, 1H), 7.11 (dd, *J* = 8.9, 2.7 Hz, 1H), 6.81 (dd, *J* = 9.8, 2.1 Hz, 1H), 6.30 (d, *J* = 2.0 Hz, 1H), 5.01(d, *J* = 2.4Hz, 2H),3.70 (t, *J* = 2.5 Hz, 1H). NMR data was in accordance with the literature.[1]

Synthesis of 4-(*N*-ethyl-*N*-(propargyloxycarbonyl)amino)-7-nitrobenzofurazan (prodye 2)

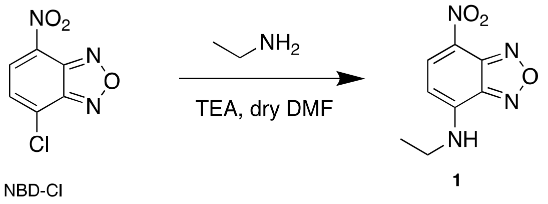

4-Chloro-7-nitro-1,2,3-benzoxadiazole (**NBD-Cl**) (73 mg, 0.366 mmol), and triethylamine (38 µL, 0.366 mmol) were dissolved in dry DMF (1.5 mL) under N_2 atmosphere. A solution of ethylamine (209 µL of 2 mol/L in THF) was then added dropwise to the mixture. The reaction mixture was stirred at room temperature for 12 h, and monitored by TLC until completion (SiO_2, MetOH 5:95 DCM). Subsequently, solvents were removed by rotary evaporation and the crude was purified *via* silica gel column chromatography EtOAc (0→40% *v*/*v*)-Hexane to yield **1** as an orange solid (70 mg, 0.336 mmol, 92 % yield); **R*f***= 0.30 (40% EtOAc in Hexane). **^{1}H NMR** (500 MHz, DMSO-d$_6$) δ = 9.50 (s, 1H), 8.49 (d, *J* = 10 Hz, 1H), 6.38 (d, *J* =10 Hz, 1H), 3.51 (s, 2H), 1.27 (t, *J* = 5 Hz, 3H) ppm. **^{13}C NMR** (126 MHz, DMSO-d$_6$) δ = 144.9, 144.4, 144.1, 120.5, 98.9, 38.2, 12.3 ppm. **HRMS (ESI) (*m/z*)**: [M]$^+$ calcd. for $C_8H_8N_4O_3$, 208.06; found, 209.06 [M + H]$^+$ and 231.06 [M + Na]$^+$.

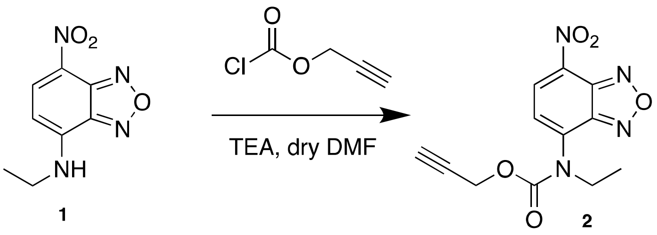

Compound **1** (67 mg, 0.322 mmol) was dissolved in dry DMF (1 mL) under N_2 atmosphere. Then, triethylamine was added (100 µL, 0.966 mmol), and after 15-30 min propargyl chloroformate (47.1 µL, 0.483 mmol) was added dropwise to the mixture. The reaction mixture was stirred at room temperature for 12 h, and monitored by TLC until completion (SiO_2, Hexane 70:30 EtOAc). Subsequently, solvents were removed by rotary evaporation. The crude was purified *via* semipreparative TLC chromatography (30% EtOAc in Hexane) to yield **2** as a yellowish solid (80 mg, 0.276 mmol, 85 % yield); **R*f***= 0.45 (30% EtOAc in Hexane). **^{1}H NMR** (500 MHz, DMSO-d$_6$) δ = 8.73 (d, *J* = 10 Hz, 1H), 7.40 (d, *J* = 5 Hz, 2H), 7.78 (d, *J* =5 Hz, 1H), 4.78 (d, *J* = 2.5 Hz, 2H), 3.94 (q, *J* = 7 Hz, 2H), 3,56 (t, *J* = 2.5 Hz, 1H), 1.18 (t, *J* = 7 Hz, 3H) ppm. **^{13}C NMR** (126 MHz, DMSO-d$_6$) δ = 152.8, 147.9, 143.7, 136.6, 134.1, 133.0, 126.5, 78.1, 78, 53.8, 45.4, 13.6 ppm. **HRMS (ESI) (*m/z*)**: [M]$^+$ calcd. for $C_{12}H_{10}N_4O_5$, 290.07; found, 291.07 [M + H]$^+$ and 313.06 [M + Na]$^+$.

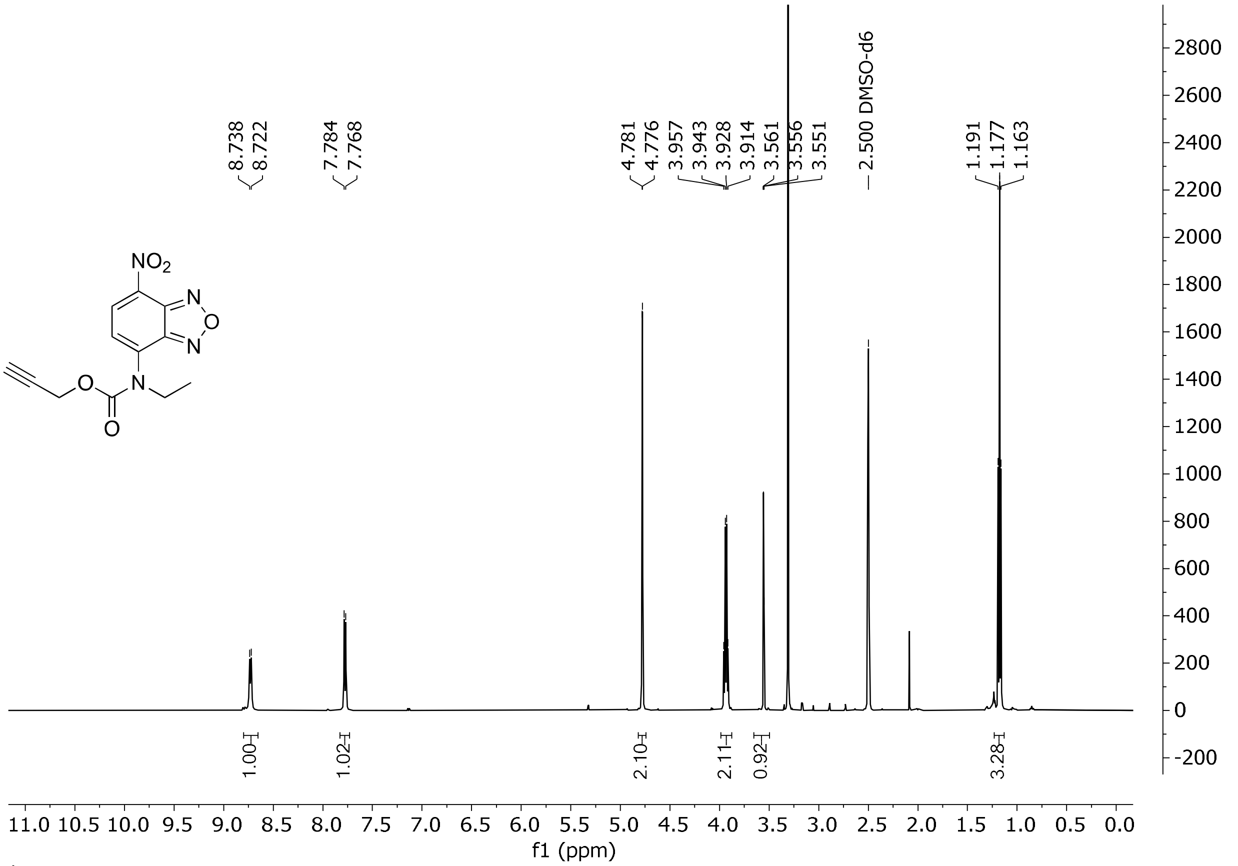

^1H NMR spectrum of compound 2. Spectra were taken in DMSO-d_6 at 500 MHz.

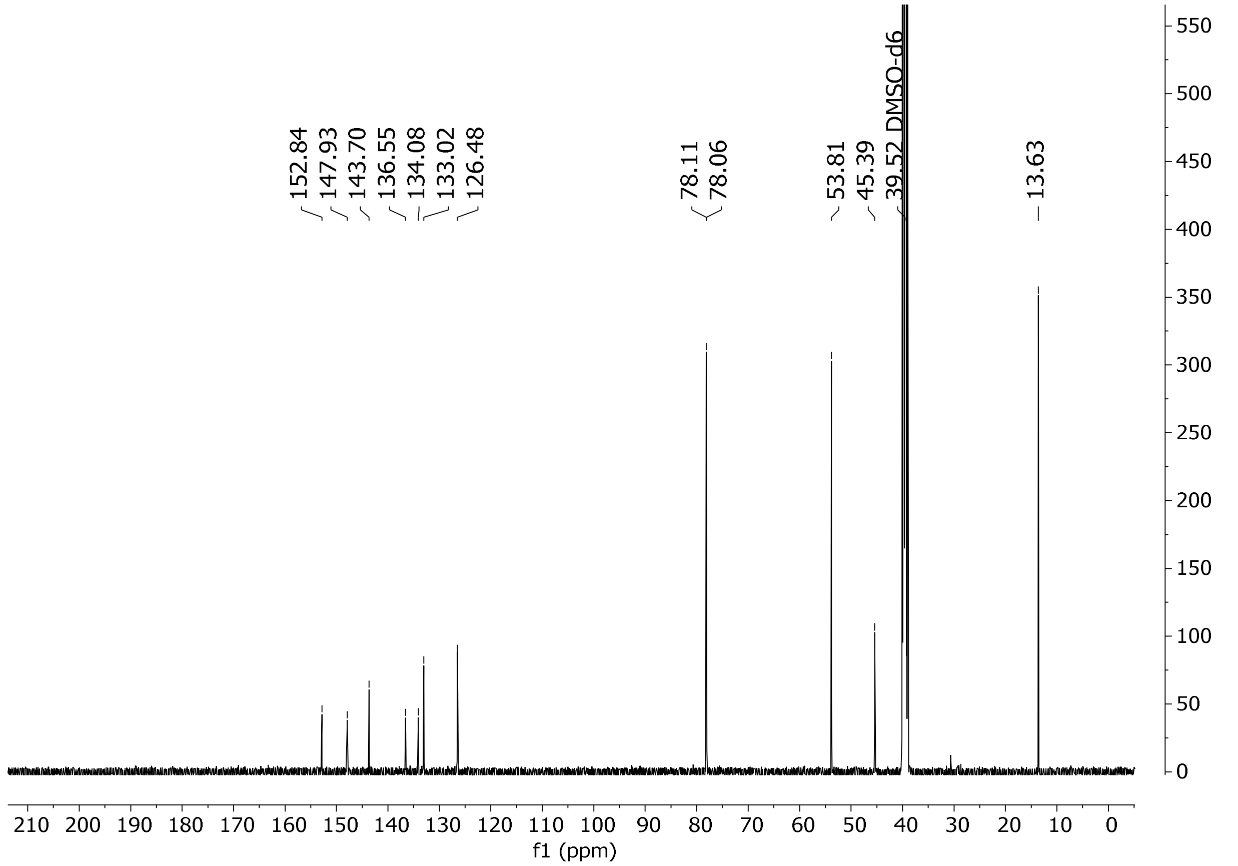

^{13}C NMR spectrum of compound 2. Spectra were taken in DMSO-d_6 at 126 MHz

Synthesis of *N*-(propargyloxycarbonyl)-fluoxetine (prodrug 4)

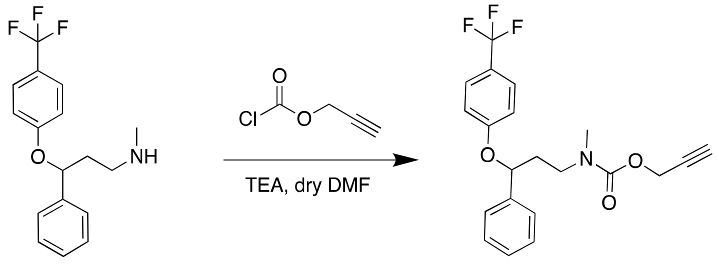

3 **4**

Fluoxetine HCl (**3**) (300 mg, 0.868 mmol), and triethylamine (242 µL, 1.74 mmol) were dissolved in dry DMF (5 mL) under N_2 atmosphere. A solution of propargyl chloroformate (84.63 µL, 0.868 mmol) was then added dropwise to the mixture. The reaction mixture was stirred at room temperature for 12 h, and monitored by TLC until completion (SiO_2, Hexane 66:33 EtOAc). Subsequently, solvents were removed by rotary evaporation, the crude dissolved in DCM (20 mL), and washed once with distilled water (15 mL), and twice with brine (15 mL). The organic layers were collected, dried over anhydrous $MgSO_4$ and concentrated *in vacuo*. The crude was purified *via* flash chromatography EtOAc (0→60% *v*/*v*)-hexane to yield **4** as yellowish white crystals (309 mg, 0.789 mmol, 91 % yield); **R*f***= 0.73 (33% EtOAc in hexane). **^{1}H NMR** (500 MHz, DMSO-d$_6$) δ = 7.54 (d, J = 9 Hz, 2H), 7.40 (dd, J^1 = 7 Hz, J^2 = 2 Hz, 2H), 7.34 (t, J =7 Hz, 2H), 7.26 (tt, J^1 = 7 Hz, J^2 = 2 Hz, 1H), 7.06 (d, J = 9 Hz, 2H), 5.45 (d, J = 4 Hz, 1H), 4.59 (m, 2H), 3.39 (m, 3H), 2.83 (s, 3H), 2.13 (dt, J = 7Hz, 1H), 2.02 (s, 1H) ppm. **^{13}C NMR** (126 MHz, DMSO-d$_6$) δ = 160.3(ArC), 154.6 (CO), 140.6 (ArC), 128.6 (ArCH), 127.8 (ArCH), 126.8 (ArCH, q, J^{CF} = 2.5 Hz), 126.0 (ArCH), 124.4 (CF_3, q, J^{CF} = 271 Hz) 121.2 (ArC, q, J^{CF} = 32 Hz), 116.1 (ArCH), 79.2 (C), 77.2 (CH_2), 76.9 (CH), 52.4 (CH_2), 45.7 (CH_2), 45.0 (CH_2), 36.1 (CH_2), 35.6 (CH_2), 34.5 (CH_2), 33.8 (CH_2) ppm. *N.b.* rotamers present in ^{1}H and ^{13}C NMR spectra. **^{19}F NMR** (470 MHz, DMSO-d$_6$) δ = -59.89 ppm. **HRMS (ESI) (*m/z*)**: $[M]^+$ calcd. for $C_{21}H_{20}F_3NO_3$, 391.14; found, 392.14820 $[M + H]^+$ and 414.12910 $[M + Na]^+$.

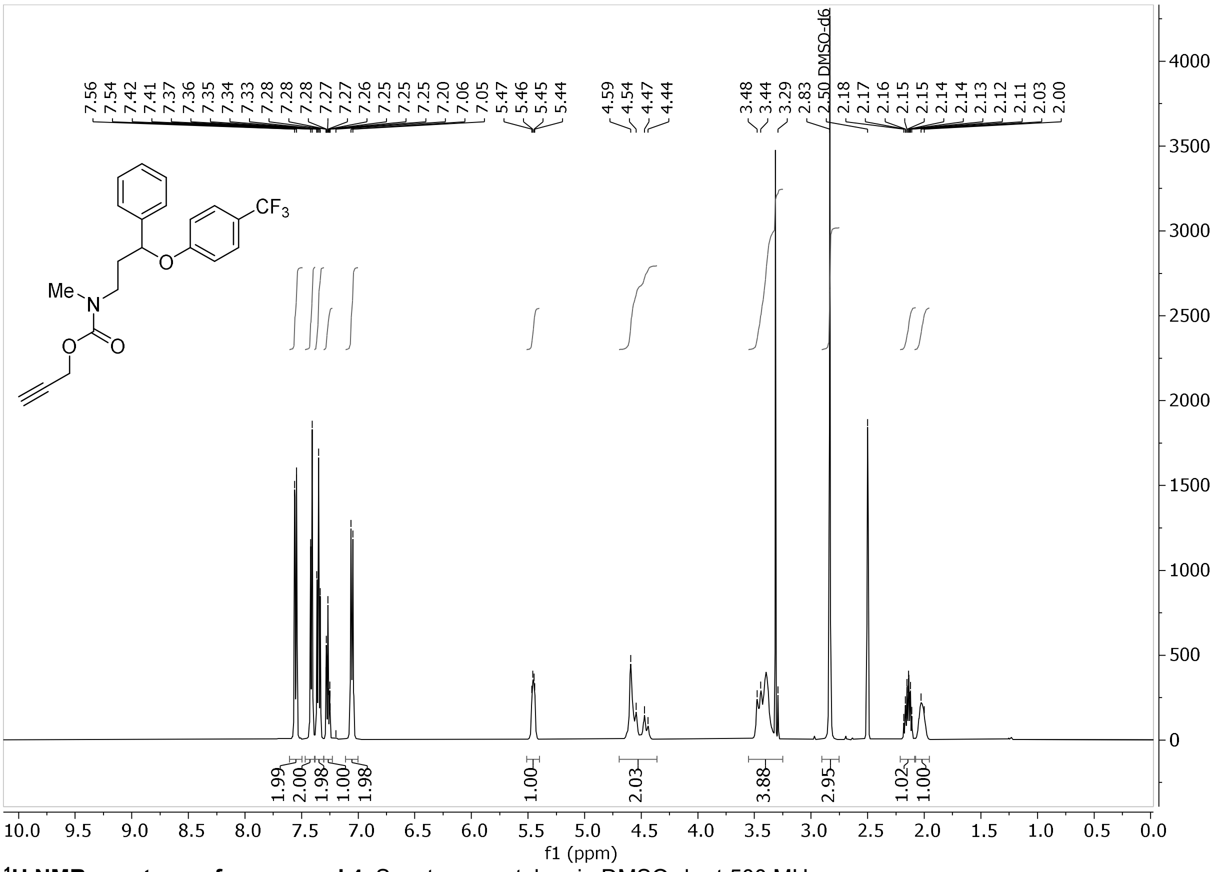

^{1}H NMR spectrum of compound 4. Spectra were taken in DMSO-d$_6$ at 500 MHz.

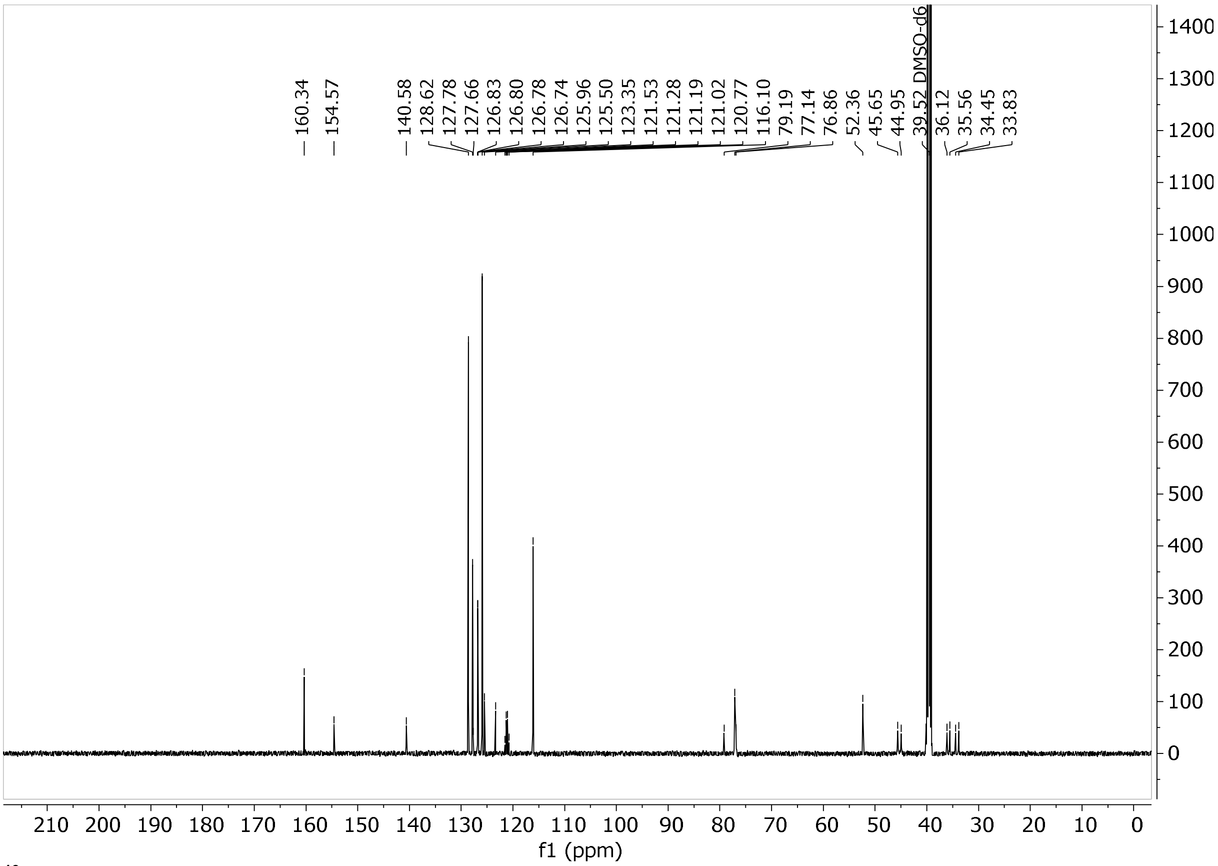

^{13}C NMR spectrum of compound 4. Spectra were taken in DMSO-d_6 at 126 MHz.

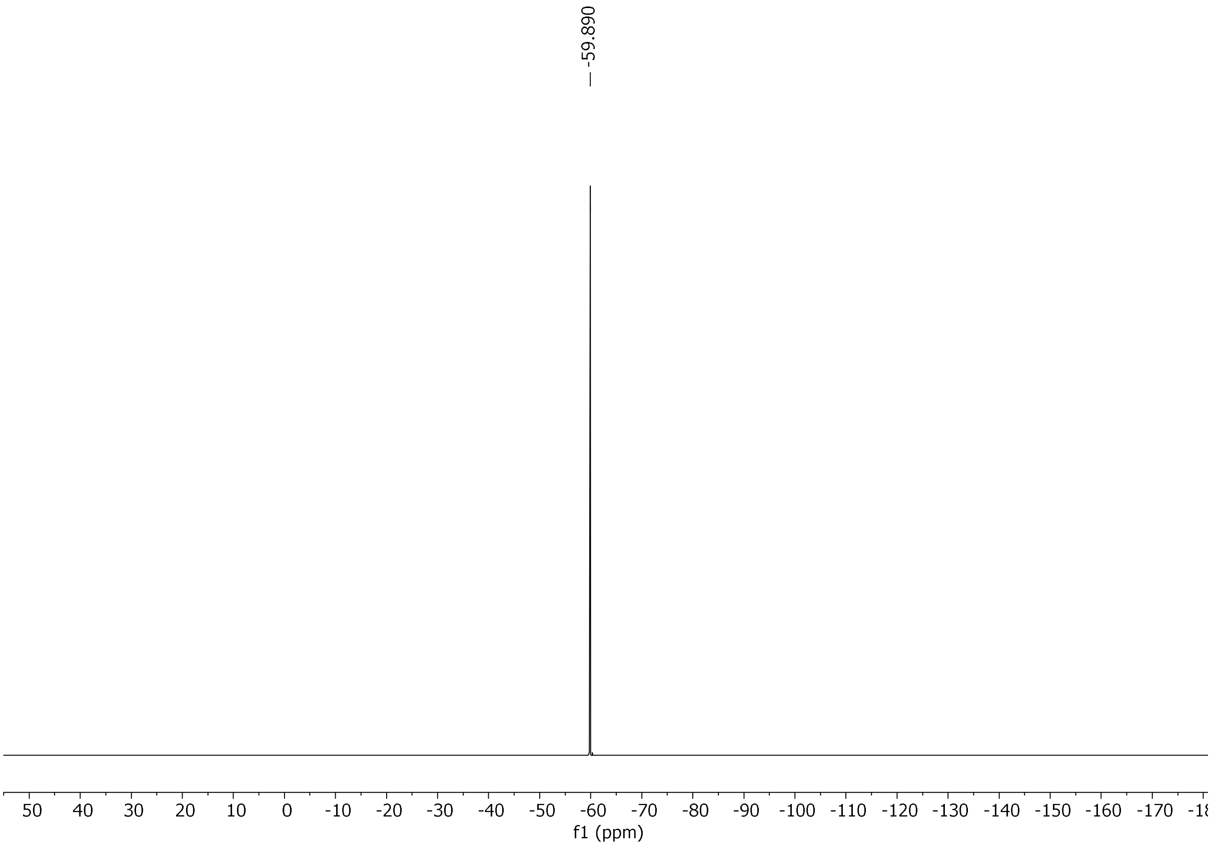

^{19}F NMR spectrum of compound 4. Spectra were taken in DMSO-d_6 at 470 MHz.

Fluorogenic reaction with naked Au-NPs using Pro-Res. 1 mL solution containing the desired concentration of **Au-NPs** (40 µg/mL) and 40 µM of **Pro-Res** was prepared in an Eppendorf tube in PBS. Reactions were shaken at 700 rpm and 37 ºC in a Thermomixer and fluorescence intensity measured at different time points in a PerkinElmer EnVision 2101 multilabel reader (Ex/Em: 550 /580 nm). Samples were repeated in triplicate. The results are shown in **Figure S1**. The concentration of resorufin product (µM) was calculated based on the standard curve of resorufin.

Fluorogenic reaction with Au-microimplants using compound prodye 2. 0.5 mL solution containing the desired concentration of **Au-microimplants** (0.05, 0.08, 0.1 and 0.5 mg/mL) and 50 µM of prodye **2** was prepared in an Eppendorf tube in PBS containing 30% of methanol (MeOH). The mixtures were shaken at 700 rpm and 37 ºC in a Thermomixer for 24 h. From this initial screen (see **Figure S4c-d**), the concentration of 0.1 mg/mL **Au-microimplants** was selected for further testing in the absence (PBS) and presence of serum (PBS + 10% FBS) using 50 µM of prodye **2**. Note that 0.1 mg/mL of **Au-microimplant** is equal to approx. 10 µM of Au (exact calculation 9.65 µM). Fluorescence intensity measured at different time points in a PerkinElmer EnVision 2101 multilabel reader (Ex/Em: 485/535 nm). The conversion values were calculated from fluorescence intensity measurements at λex/em = 485/535 nm using the fluorescence intensity of **1** (50 µM) as 100%. Negative controls: **2** (50 µM) with or without Au-implants (0.1 mg/mL). Each experiment was performed at least in triplicate, and the values given correspond to the mean value ± SD of $n \geq 3$. Naked **Au-NPs** were tested alongside to determine the effect of serum on freestanding and polymer-supported **Au-NPs**. The results are shown in the main manuscript and in **Figure S4c-e.**

Recyclability study of Au-microimplants using prodye 2. Au-microimplants (0.1 mg) were added to a 1 mL solution of **2** at 50 µM in PBS or serum (PBS + 10% of FBS). The mixtures were shaken at 700 rpm and 37ºC in a Thermomixer and reactions fluorescence intensity measured at 24 h by in a PerkinElmer EnVision 2101 multilabel reader (Ex/Em: 485/535 nm). Au-microimplants (0.1 mg) were recovered by centrifugation (8000 rpm, 5 min) and washed with distilled water. A freshly-prepared solution of **2** at 50 µM in PBS or serum as appropriate was added to the **Au-microimplants** and the mixtures shaken at 700 rpm and 37ºC. After 24 h, 30% MeOH was added to the vials and the fluorescence of the supernatants were measured. This cycle was repeated 10 times. The results are shown in **Figure S5.**

Fluorogenic reaction with naked Au-NPs and Au-microimplant using Pro-Res in presence of glucose and ascorbate. 1 mL solution containing the desired concentration of **Au-NPs** (40 µg/mL) or 0.1 mg/mL of **Au-microimplants** and 40 µM of **Pro-Res** containing different concentration of glucose (0, 0.01, 0.05, 0.1, 0.5, 1, 2.5, 10 and 15 mM) or ascorbic acid (0, 10, 25, 50, 100 and 150 µM), as appropriate, were prepared in Eppendorf tubes in PBS. Reactions were shaken at 700 rpm and 37 ºC in a Thermomixer and fluorescence intensity measured at different time points in a PerkinElmer EnVision 2101 multilabel reader (Ex/Em: 550 /580 nm). Samples were repeated in triplicate. The results are shown in **Figure S6a-d**. The concentration of resorufin product (µM) was calculated based on the standard curve of resorufin.

Fluorogenic reaction with Au-microimplant using Pro-Res at different pH in phosphate buffers. 1 mL solution containing the desired concentration of 0.1 mg/mL of **Au-microimplants** and 40 µM of **Pro-Res** containing 0.1 M phosphate buffers at different pH (5.8, 6.6, 7, 7.4, 8) were prepared in Eppendorf tubes in PBS. Reactions were shaken at 700 rpm and 37 ºC in a Thermomixer and fluorescence intensity measured at different time points in a PerkinElmer EnVision 2101 multilabel reader (Ex/Em: 550 /580 nm). Samples were repeated in triplicate.The results are shown in **Figure S6e**. The concentration of resorufin product (µM) was calculated based on the standard curve of resorufin.

Fluorogenic reaction with naked Au-NPs and Au-microimplants using pro-dye 2 in the presence of biogenic thiols. 0.5 mL solution with 0.1 mg/mL of **Au-microimplants** or 40 µg/mL of **naked Au-NPs** and 50 µM of prodye **2** containing different concentrations (0, 10, 25, 50, 70, and 100 µM) of glutathione or cysteine were prepared in Eppendorf tubes in PBS. The mixtures were shaken at 700 rpm and 37 ºC in a Thermomixer. After 3h, 12h and 24h, 30% MeOH was added to each vials and the fluorescence of the supernatants were measured. Fluorescence intensity measured at different time points in a PerkinElmer EnVision 2101 multilabel reader (Ex/Em: 485/535 nm). The conversion values were calculated from fluorescence intensity measurements at λex/em = 485/535 nm using the fluorescence intensity of **1** (50 µM) as 100%. Negative controls: **2** (50 µM) with or without **Au-microimplants** (0.1 mg/mL) or **Au-NPs** (40 µg/mL). Each experiment was performed at least in triplicate, and the values given correspond to the mean value ± SD of $n \geq 3$. The results are shown in **Figure S7**.

Prodrug-into-drug conversion study. Prodrug **4** (4 µL, 150 mM stock solution in DMSO) was added to a H_2O:MeOH 7:3 solution (496 mL of the reaction medium) in a 1 mL Eppendorf, containing 1 mg / mL of **Au-microimplants**. Reactions were carried out for 0, 18, 24, 32 and 44 hours with continuous stirring (700 rpm) at 37 ºC using a Thermomixer. Afterwards, the **Au-microimplants** were collected by centrifugation (5 min at 8000 rpm) and the supernatant was analyzed by LC/MS (Agilent 1260 Infinity II) using an ELSD detector. Fluoxetine **3** (1.2 mM) was dissolved in H_2O:MeOH 7:3 solution (0.5 mL) in the same conditions as described above and analyzed as a positive control. Each experiment was performed at least in triplicate, and the values given correspond to the mean value ± SD of $n \geq 3$. Each measurement was taken from distinct samples. R^2 is the coefficient of determination, used as statistical parameter of goodness of fit in the calibration curves. Data analysis was performed using *OriginPro 8* statistical software. The results are shown in **Figure S12-13.**

3. Biological Studies

Cell culture. Lung adenocarcinoma A549 cells (a kind gift from Dr Wilkinson) and neuroblastoma SH-SY5Y cells (a kind gift from Prof. Kathryn Ball) were cultured in Dulbecco's Modified Eagle Media (DMEM) supplemented with serum (10 % of FBS) and L-glutamine (2 mM). Each cell line was checked for mycoplasma before use and maintained in normoxic conditions at 37ºC and 5% CO_2. Cells were seeded in a 96-well plate at 1000 cells/well for A549 or 7500 cell/wells for SH-SY5Y and incubated for 24 h before treatment.

Study of the biocompatibility of Au-microimplants. The tolerability of cells to **Au-microimplants** was tested by performing dose-response studies in A549 and SH-SY5Y cells. Cells were plated as indicated above. Each well was then replaced with 100 µL of fresh media containing **Au-microimplants** at 0.6, 0.8, 1 and 1.2 mg/mL for both cells. After 1 week, PrestoBlue™ cell viability reagent (10 % *v*/*v*) was added to each well and the plate incubated for 90 min. Fluorescence emission was detected using a PerkinElmer EnVision 2101 multilabel reader (Ex/Em: 540/590 nm). Experiments were performed in triplicate. All conditions were normalized to the untreated cells (100 %). The results are shown in **Figure S8 a-b.**

Cell viability study of the drug and pro-drug. A549 and SH-SY5Y cells were plated as indicated above. The corresponding wells were then replaced with a solution of compound **3** or **4** at different concentrations (2.5, 10, 25, 50, 100 and 150 µM) containing 0.1% *v*/*v* of DMSO. Untreated cells were incubated with DMSO (0.1% *v*/*v*). After 1 week, PrestoBlue™ cell viability reagent (10 % *v*/*v*) was added to each well and the plate incubated for 90 min. Fluorescence emission was detected using a PerkinElmer EnVision 2101 multilabel reader (Ex/Em: 540/590 nm). Experiments were performed in triplicate.

Of note, see **Figure S8c**, there was a minor but significant reduction of cell proliferation after treatment with Fluoxetine **3**. This is in agreement with a recent study that reports anticancer properties for **2**.[7] This effect was not observed in cells treated with prodrug **4**, which further demonstrates the inactivation of **4**.

Cell-based 5-HT2B agonist assay. The agonist bioactivity of **3** and **4** for the 5-HT2B receptor was determined in cells using a FLIPR® Calcium 5 Assay (Molecular devices, Inc.). Experiments were performed by Reaction Biology (US) in cooperation partner with PharmaCore Labs (China). Cells over-expressing the 5-HT2B receptor were seeded in black, clear-bottomed well half area plates in 50 µl volume and incubated for 16-24h. Next day, media (Ham's F12 containing 10 % FBS, 200 µg/mL Zeocin™) was removed from cell plates and dye (Calcium 5 Assay Reagent) solution prepared in assay buffer (1.26 mM 1X HBSS, 20 mM HEPES) was added. Then, the cell plates were incubated at 37 ºC and 5% CO_2 for 1 h. Compounds **3** and **4** were tested at 100 µM starting concentration with a 5-fold serial dilution. Compounds in 100 % DMSO stock solution were diluted in assay buffer. 30 µl of the serial dilutions were then added to the cell plate. After incubation with the dye, for agonist testing the plates were placed in the Fluorescent Imaging Plate Reader (FLIPR® instrument). The calcium flux signal was monitored for 5-6 min. Assay buffer used for the negative control wells contained the same final concentration of DMSO as was present in the wells containing the test compounds. BW-723C86 drug was used such as a positive control. The results are shown in **Figure S11.**

4. In Vivo Experiments

Zebrafish husbandry. Animals were bred and raised at the Queen's Medical Research Institute, University of Edinburgh, in accordance with the Animals in Scientific Procedures Act 1986 and under British Home Office project licence 70/8805. Embryos were obtained through natural spawning from the adult WIK wild type zebrafish line.[8] Embryos were maintained at low density (~40/50 mL zebrafish water) at 28 °C with a 14h light/10h dark cycle. For implant studies, on reaching larval stage 3 days post-fertilisation (dpf), animals were implanted with resins at room temperature then maintained as before until 5 dpf.

Implantation procedure. Implantation of **Au-microimplants** (catalytic devices) or **OH-microimplants** (catalytically inactive; negative control) was carried out at 3 dpf. Larvae were anaesthetised with 2.5 mM ethyl 3-aminobenzoate methanesulfonate (Tricaine) in zebrafish water (pH 7.2) before being immobilised in 2% (w/v) low melting point agarose (in zebrafish water). The head was exposed, and an incision made in the skin between the developing eye cups using an optical surgery scalpel. Implants (60-75 μm in diameter) were positioned inside the incision using custom-prepared glass needles pulled from borosilicate glass. Larvae were released from the agarose and allowed to recover in zebrafish water for 1 h before being incubated in drugs of interest at 28 °C on a 14 h light/ 10 h dark cycle until 5 dpf. Full procedure of the implantation method is shown in **Figure S9.**

Confocal studies using prodye 2. **Au-microimplants** were implanted as described and larvae incubated in 1% DMSO (negative control) or with 2.5 µM of **2** in 1% DMSO for ~44h. The zefrafish were then imaged using a Olympus FV3000 Confocal Laser Scanning Microscope using a 20x objetive. The setting of the confocal microscope were as follows: λ_{exc}= 488 nm and λ_{em}= 514-553 nm.

Incubation for behavioral studies. In initial trials, without implants, control recordings were obtained in 1% DMSO in zebrafish water. Larvae were then pre-incubated in drug of interest in 1% DMSO for 2h prior to treatment recording. For **Au-microimplants** and **control microimplants** trials, devices were implanted as described and larvae incubated in 1% DMSO or prodrug **4** in 1% DMSO for ~44h.

Behavioural assay and data acquisition. Spontaneous swimming behaviour of 5 dpf larvae was observed in each treatment group. Larvae were acclimatised to room temperature for 1h prior to recording and screened for normal developmental appearance.[9] For recordings with implants, larvae in which the implant was not centred in the head were discarded. Individual larval swimming was tracked in a cell culture dish (35mm x 10mm) using EthoVision XT 7 software (Noldus Information Technology) via a Sony ExwaveHAD B&W video camera. The preparation was lit from below by a light box. Recordings were of 10 min duration in each condition. Acquisition of total distance (mm) and mean and maximum speed (mm s^{-1}) was automatically performed by the software. Swimming parameter data are reported ± standard error of the mean (SEM). All statistical analysis was carried out on raw data. Data were assessed for normality and analysed with appropriate parametric or non-parametric tests as described in the results. All statistical tests were performed using GraphPad Prism 8.3 (GraphPad Software, LLC); ns – no significance, *P<.05, **P<.01, ***P<.001, ****P<.0001. Where data are presented as percentage of control in the text, the raw data are also stated.

SUMMARY OF RESULTS:

Influence of psychotropic drugs in larval zebrafish behavior**.** To select compounds for further study, initial trials were performed in intact larvae (no implants). Distance travelled and speed of spontaneous larval swimming in a 10-minute window was measured to determine the effects of NMDA, GABA and Fluoxetine **3** exposure on normal behaviour. All solutions were prepared in 1% DMSO in zebrafish water. Following control recordings in 1% DMSO, 5 dpf larvae were treated with 150 μM GABA, 100 μM NMDA or 50 μM Fluoxetine **3** for 2 h (see **Figure S10**). 2 h incubation in 100 μM NMDA had no effect on measured swimming parameters (*Wilcoxon Signed-Ranks test: P=.8408; n=20*). 150 μM GABA produced a decrease in average speed (2.90mms^{-1} ± 0.26 mms^{-1} to 2.04 mms^{-1} ± 0.28 mms^{-1}; *paired t test: t(19)=2.169, P=0.0430; n=20*) and a small but insignificant decrease in distance (*t(19) = 2.039, P=.0556*). 50 μM Fluoxetine **3** significantly altered both swimming parameters, as described below and was thus selected for further investigation.

Prodrug **4** does not elicit a reduction in larval swim distance and speed. Distance travelled and speed of spontaneous larval swimming in a 10-minute window was measured to determine the effects of **4** exposure on normal behaviour. Larvae were treated with 50 μM drug **3** or prodrug **4** in solution with 1% DMSO in zebrafish water. Control recordings were made in 1% DMSO in zebrafish water. As shown in **Figure S11**, 2 h incubation in 50 μM drug **3** reduced mean total distance travelled from 2285mm ± 142.70mm to 859.50mm ± 92.01mm (paired t test: *t(19) = 11.49, P<.0001; n=20*) and mean swim speed from 3.81mms^{-1} ± 0.24 mms^{-1} to 1.43 mms^{-1} ± 0.15 mms^{-1} (*t(19) = 11.5049, P<.0001*). Treatment with 50 μM prodrug **4** had no effect on these swimming parameters (Wilcoxon Signed-Ranks test: *P=0.4304*). These data indicate that drug **3** can modulate zebrafish larval swimming behaviour, and that its propargyl carbamate derivative **4** is effective in preventing its action *in vivo*.

Bioorthogonal intracranial release of **3** by Au-implants is effective in modulating zebrafish larval swimming. To confirm the capacity of **Au-microimplants** to influence larval locomotor activity via biorthogonal release of **3**, larvae were implanted with a single **Au-microimplant** at 3dpf and incubated in 50 μM prodrug **4** in 1% DMSO for 44 h. **Au-microimplants** alone had no effect on measured parameters in 1% DMSO (Kruskal-Wallis test: *P>.999;* n=22), see **Figure S14**. In the presence of **Au-microimplants**, 50 μM prodrug **4** reduced mean total distance (1071mm ± 149.9mm) to 50.34% ± 6.92% of that measured in **Au-microimplant** DMSO controls

(1911mm ± 208.8mm; *P=.0071; n=22*). Mean swim speed (1.79 mms^{-1} ± 0.25 mms^{-1}) was 53.53% ± 7.52% **Au-microimplant** DMSO control values (3.19 mms^{-1} ± 0.35 mms^{-1}; *P =.0077*). Comparison of the de-protection results with those from the initial 'drug only' assay indicates a similarity of effect in each scenario (Welch's t-test: mean total distance *t(34.40)= 1.201, P=0.2378*; mean swim speed *t(34.18)= 1.208, P=0.2354*). These data provide evidence for Au-mediated intracranial bioorthogonal activation of prodrug **4** that is sufficient to influence larval zebrafish swimming comparable with treatment with drug **3** at 50 μM.

OH-microimplants are ineffective in deprotecting prodrug **4** *in vivo.* Behavioural assays were repeated in zebrafish larvae grafted with carboxyl-functionalized Tentagel resins (**OH-microimplants**) to confirm that deprotection of prodrug **4** in vivo requires the presence of Au, see **Figure S14c-d**. Neither mean total distance (Welch's t-test: *t(40.94) = 0.943, P=0.3514; n=22*) nor mean speed of **OH-implant** larvae was influenced by 44 h incubation in prodrug **4** (*t(40.94) = 0.942, P=0.3519*).

5. Supplementary References

[1] M. Sancho-Albero, B. Rubio-Ruiz, A. M. Perez-Lopez, V. Sebastian, P. Martin-Duque, M. Arruebo, J. Santamaria, A. Unciti-Broceta, *Nature Catalysis* **2019**, *2*, 864-872.
[2] G. Frens, *Nature-Physical Science* **1973**, *241*, 20-22.
[3] J. Piella, N. G. Bastus, V. Puntes, *Chemistry of Materials* **2016**, *28*, 1066-1075.
[4] D. G. Duff, A. Baiker, P. P. Edwards, *Langmuir* **1993**, *9*, 2301-2309.
[5] N. A. Heutz, P. Dolcet, A. Birkner, M. Casarin, K. Merz, S. Gialanella, S. Gross, *Nanoscale* **2013**, *5*, 10534-10541.
[6] M. C. Ortega-Liebana, J. Bonet-Aleta, J. L. Hueso, J. Santamaria, *Catalysts* **2020**, *10*.
[7] J. Bi, A. Khan, J. Tang, S. Wu, W. Zhang, R. C. Gimple, T. Koga, A. M. Armando, S. Miki, H. Yang, B. Prager, E. J. Curtis, D. A. Wainwright, F. B. Furnari, J. N. Rich, T. F. Cloughesy, O. Quehenberger, H. I. Kornblum, A. Rzhetsky, B. F. Cravatt, P. S. Mischel. *BioRxiv,* doi.org/10.1101/2021.03.16.435487, **2021**.
[8] S. L. Johnson, L. I. Zon, *Methods in Cell Biology, Vol 60* **1999**, *60*, 357-359.
[9] D. M. Parichy, M. R. Elizondo, M. G. Mills, T. N. Gordon, R. E. Engeszer, *Developmental Dynamics* **2009**, *238*, 2975-3015.

6. Supplementary Tables and Figures

Table S1. Synthesis of citrate-based and THPC-based Au-NPs with different sizes.

Au NPs	Synthetic methods	Au:citrate	Size	UV-vis (max. peak)	Pro-Res activation (%)#
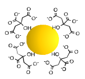	Prepared as previously described,[2] using **sodium citrate (SC)** for the reduction of **$HAuCl_4$** in water.	0.66	**15 nm**	at 518 nm	50 %
		0.88	**25 nm**	at 525 nm	47 %
		1.32	**42 nm**	at 529 nm	27 %
		2.19	**72 nm**	at 529 nm	23 %
		3.14	**97 nm**	at 533 nm	16 %
		4.11	**147 nm**	at 560 nm	13 %
**Au NPs**	**Synthetic methods**	**THPC:NaOH**	**Size**	**UV-vis (max. peak)**	**Pro-Res activation (%)#**
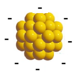	Prepared as described above, using **THPC** for the reduction of **$HAuCl_4$** in water under basic conditions.	85mM:0.2M	**2.9 nm**	at 508 nm	100 %
		76.5mM:0.17M	**9.6 nm**	at 510 nm	87 %
		68mM:0.13M	**19.7 nm**	at 522 nm	20 %
		64mM:0.1M	**25.4 nm**	at 532 nm	2 %

Catalytic properties of citrate-based Au-NPs. % of conversion of non-fluorescent **Pro-Res** (50 µM) to highly pink fluorescent resorufin by reaction with high concentration of **NPs** (94 µg/mL) in PBS at 37°C for 24h.

Table S2. Preparation methods and characteristics of Au-NP-1, Au-NP-2 and Au-NP-3.

Au NPs	Synthetic methods	DLS size distribution	UV-vis (max. peak)	ζ Potential	HAADF-STEM images
**Au-NP-1** 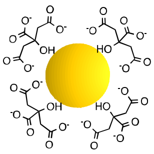	Prepared as previously described,[2] using **sodium citrate (SC)** for the reduction of **$HAuCl_4$** in water.	**23 ± 0.75 nm**	at 525 nm	-20.9 ± 0.6 mV	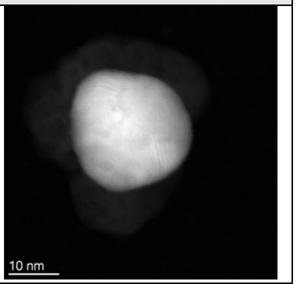
**Au-NP-2** 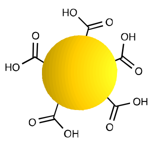	Prepared as previously described,[3] using **SC** and **tannic acid (TA)** for the reduction of **$HAuCl_4$** in water.	**5.2 ± 0.27 nm**	at 518 nm	-24.9 ± 4.5 mV	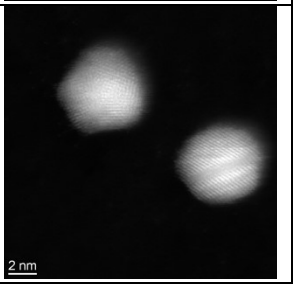
**Au-NP-3** 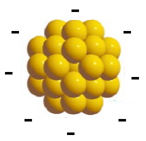	Prepared as described above, using **THPC** for the reduction of **$HAuCl_4$** in water.	**2.9 ± 0.25 nm**	at 508 nm	-19.9 ± 4.2 mV	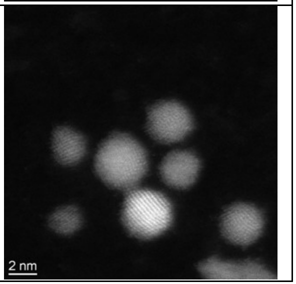

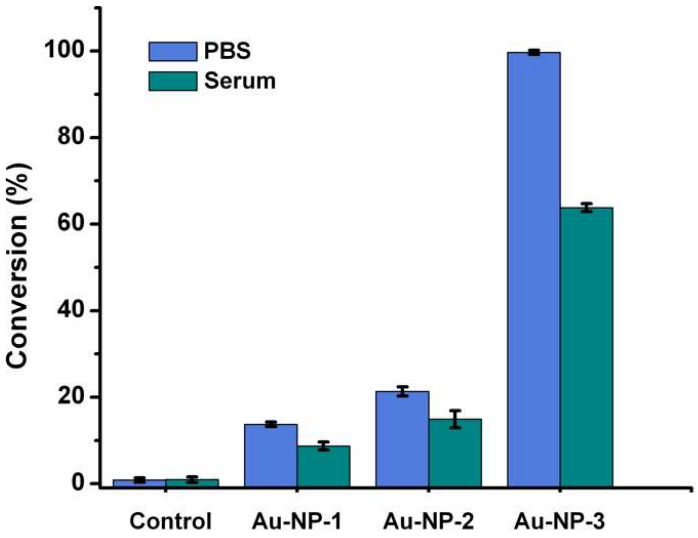

Figure S1. Catalytic properties of Au-NPs. Conversion of non-fluorescent **Pro-Res** (40 µM) to highly fluorescent resorufin mediated by the different **Au-NPs** (40 µg/mL) after 24h under biocompatible conditions (37°C, PBS or serum, pH 7.4).

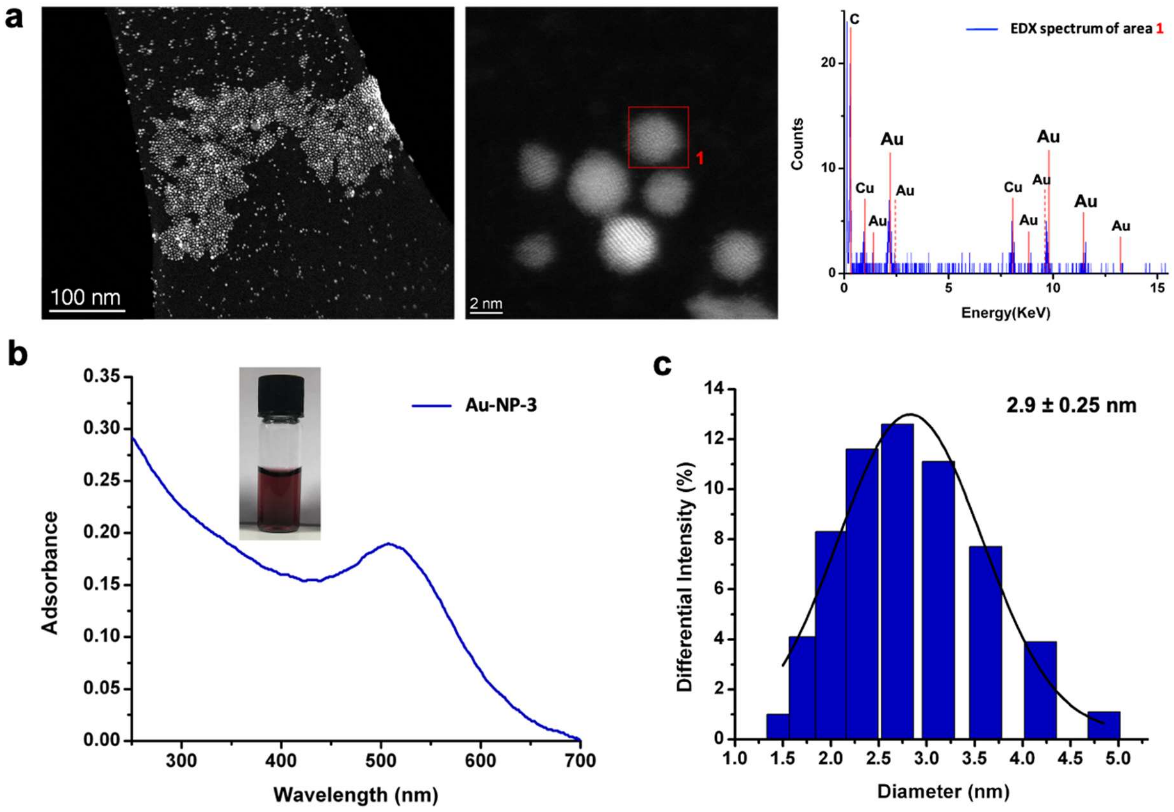

Figure S2. Full characterization of Au-NP-3. a) HAADF-STEM images of the **Au-NP-3** at different magnifications and energy-dispersive X-ray (EDX) spectra of highlighted area 1. **b)** Picture of the dispersion and UV-Visible absorption spectra of as-synthesized **Au-NP-3**. UV-vis absorption spectrum of **Au-NP-3** showing a maximum surface plasmon resonance peak at approx. 508 nm. **b)** DLS size distribution of **Au-NP-3**.

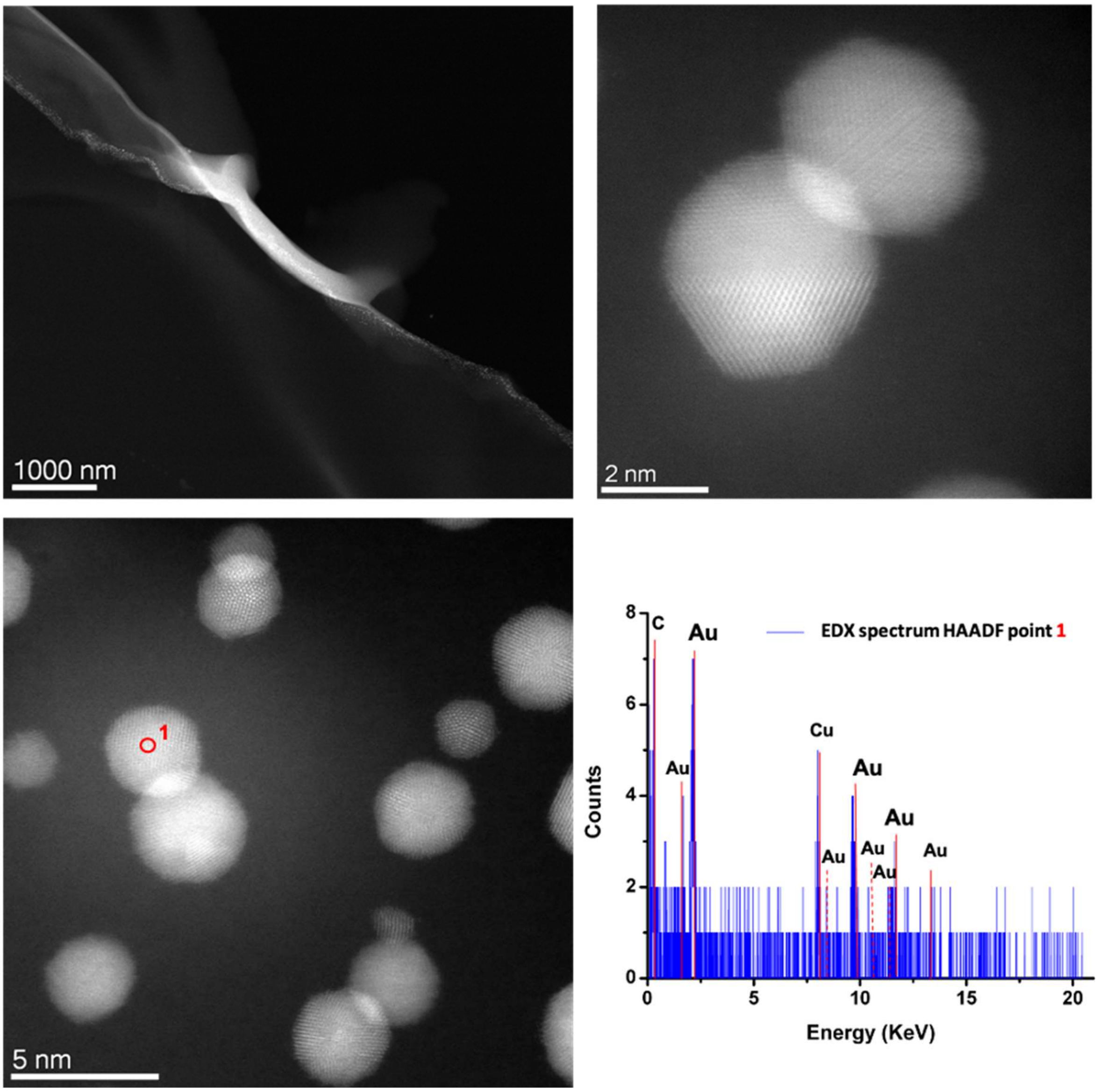

Figure S3. HAADF-STEM characterization. HAADF-STEM images of a cross-section of an **Au-microimplant** at different magnifications and energy-dispersive X-ray (EDX) spectra of highlighted point 1.

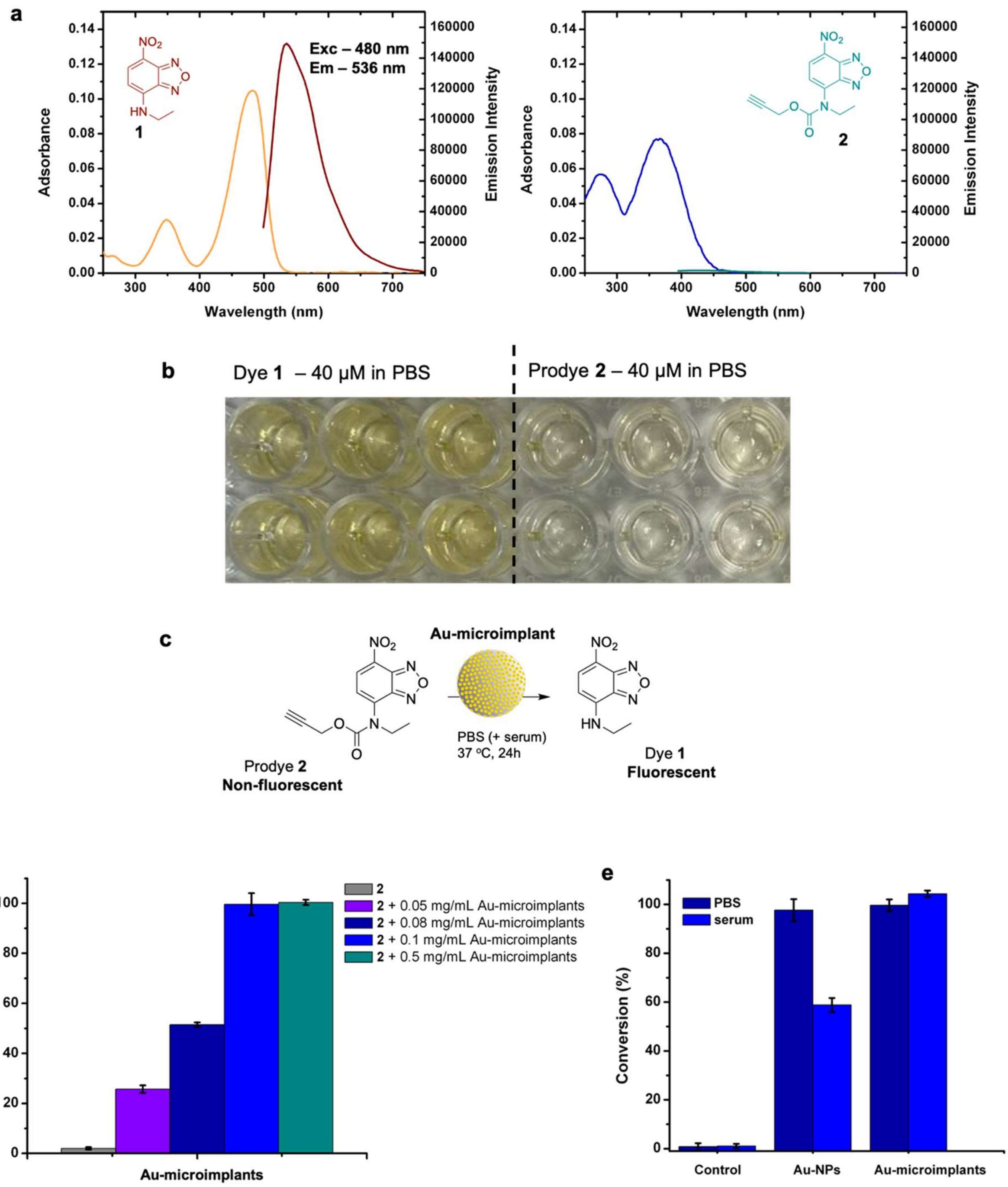

Figure S4. Study of fluorescent properties of compound 1 and 2. **a)** Absorption and fluorescence spectra of dye **1** and prodye **2** (40 uM) in PBS. **b)** Well plate with **1** and **2** solution (40 uM) in PBS. **Study of the catalytic properties of Au-microimplants. c)** Reaction of non-fluorescent prodye **2** and **Au-microimplants** to give highly fluorescent resorufin under biocompatible conditions (37°C, PBS, pH 7.4). **d)** Conversion rates (in %) after 24 h incubation of **2** (50 µM) with **Au-imicromplants** (0.05, 0.08, 0.1 and 0.5 mg/mL). The error bars are ± SD (n = 3). **e)** Comparative study of the conversion efficiencies after 24 h incubation of **2** (50 µM) with freestanding **Au-NPs** (49 µg/mL) and **Au-microimplants** (0.1 mg/mL) in PBS and PBS + 10% FBS (serum). The conversion values were calculated from fluorescence intensity measurements at λex/em = 485/535 nm using the fluorescence intensity of **1** (50 µM) as 100%. Negative controls: **2** (50 µM) without catalysts. The error bars are ± SD (n = 3).

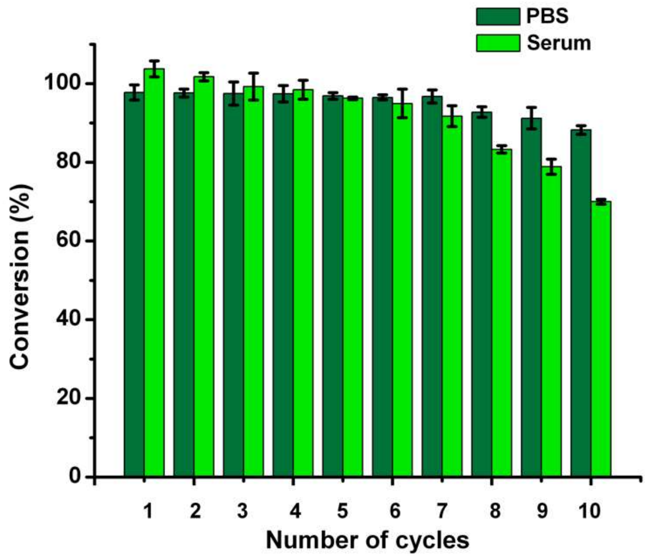

Figure S5: 10-cycle recycling test. Au-microimplants (0.1 mg/mL) were recovered after each reaction cycle and re-used in PBS and serum. Conversion (%) was measured at 24 h. The error bars are ± SD (n = 6).

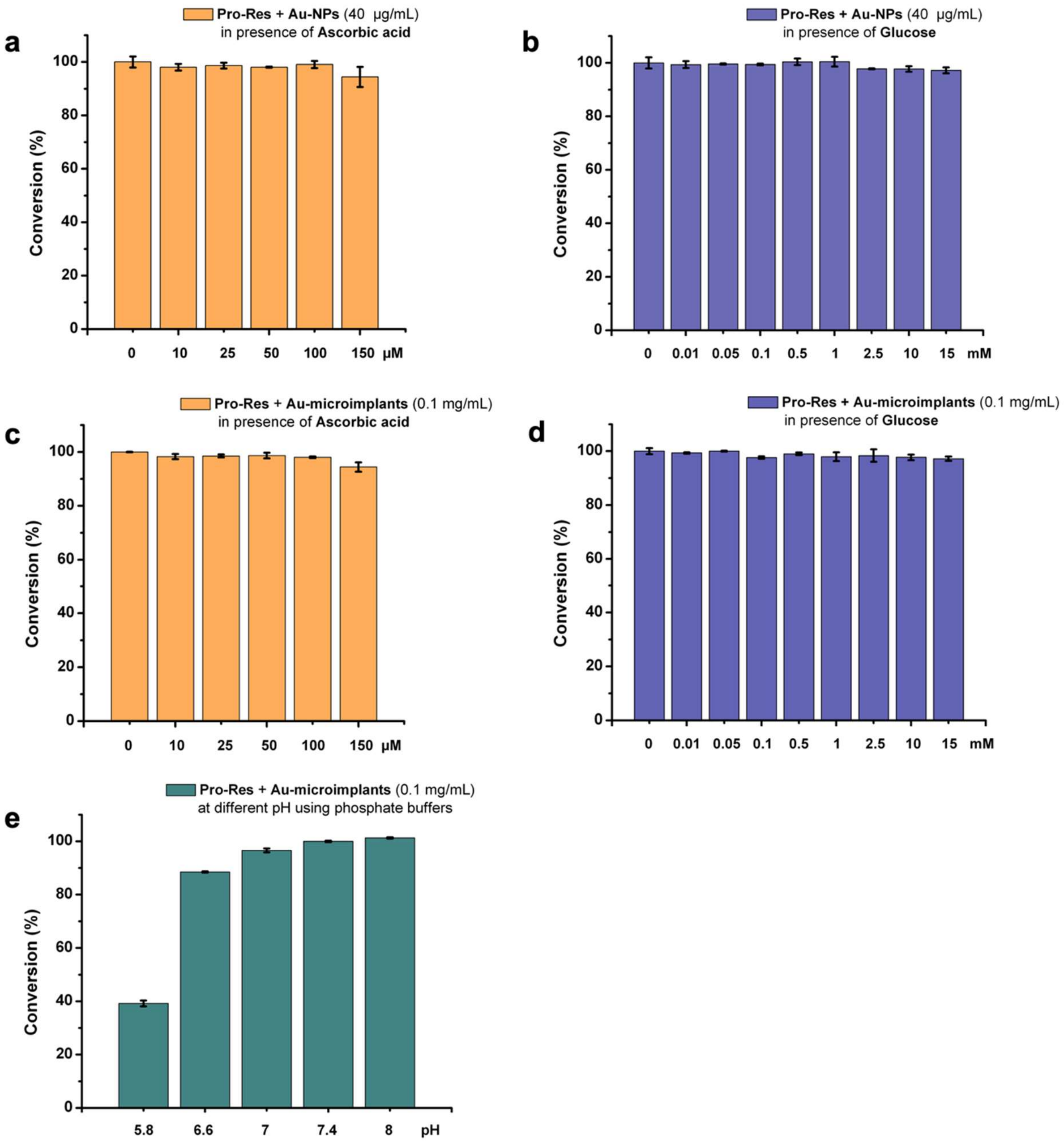

Figure S6. Comparative study of the conversion efficiencies of **Au-NPs** after 24 h incubation of **Pro-Res** (40 μM) with naked **Au-NPs** (40 μg/mL) in PBS in the presence of ascorbic acid (AA) (**a**) or glucose (**b**). Comparative study of the conversion efficiencies of **Au-microimplants** after 24 h incubation of **Pro-Res** (40 μM) with **Au-microimplants** (0.1 mg/mL) in PBS in the presence of ascorbic acid (AA) (**c**) or glucose (**d**); or different pH (5.8, 6.6, 7, 7.4, 8) using phosphate buffers (**e**). Negative controls: **Pro-Res** (40 μM) without catalysts. The error bars are ± SD (n = 3).

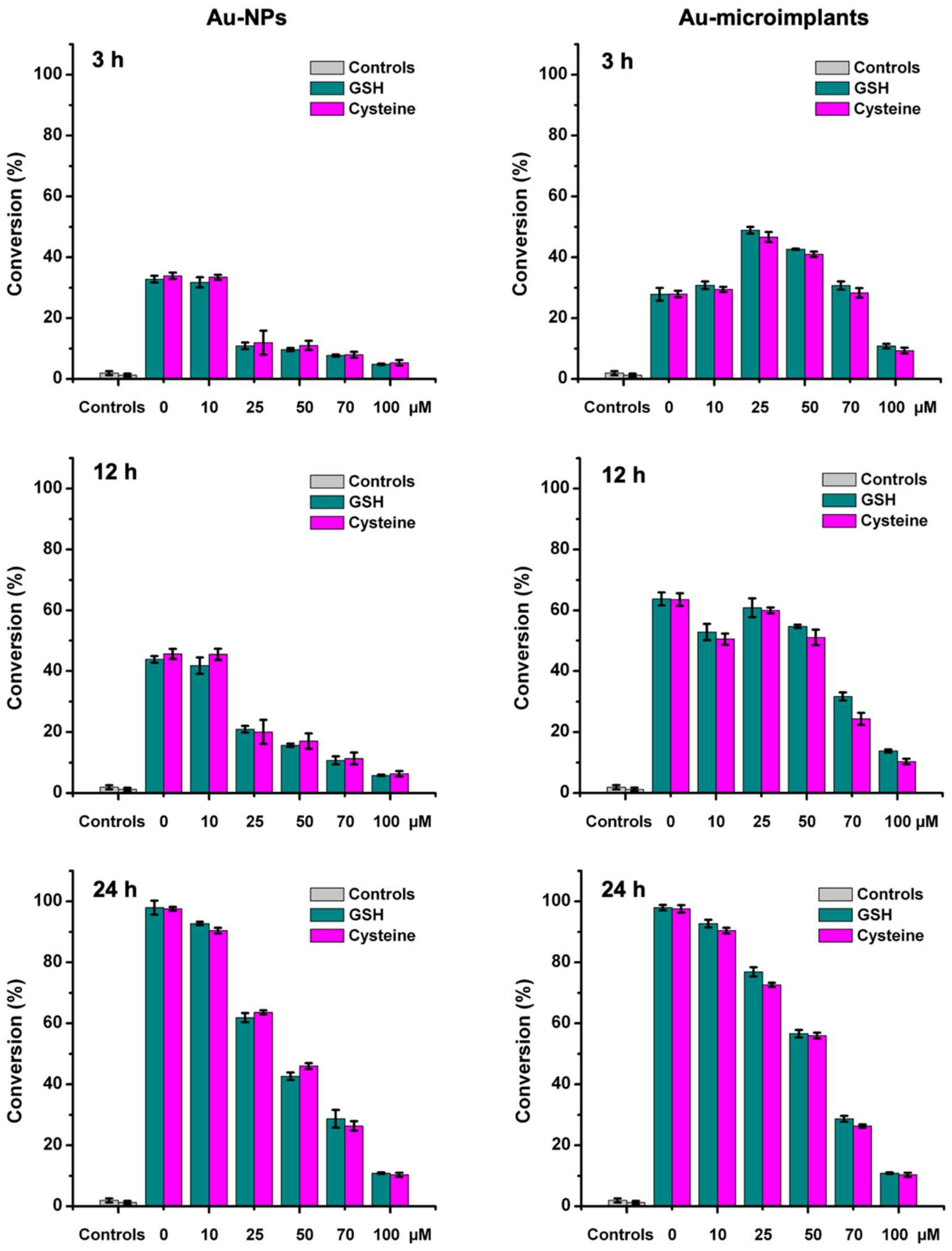

Figure S7: Comparative study of the conversion efficiencies after 3, 12 and 24 h incubation of prodye **2** (50 µM) with naked **Au-NPs** (40 µg/mL ≈ 200 µM in Au) and **Au-microimplants** (0.1 mg/mL ≈ 9.6 µM in Au) in PBS in the presence of different concentrations (0, 10, 25, 50, 70 and 100 µM) of glutathione (GSH) and cysteine. The conversion values were calculated from fluorescence intensity measurements at λex/em = 485/535 nm using the fluorescence intensity of **1** (50 µM) as 100%. Negative controls: **2** (50 µM) without catalysts. The error bars are ± SD (n = 3).

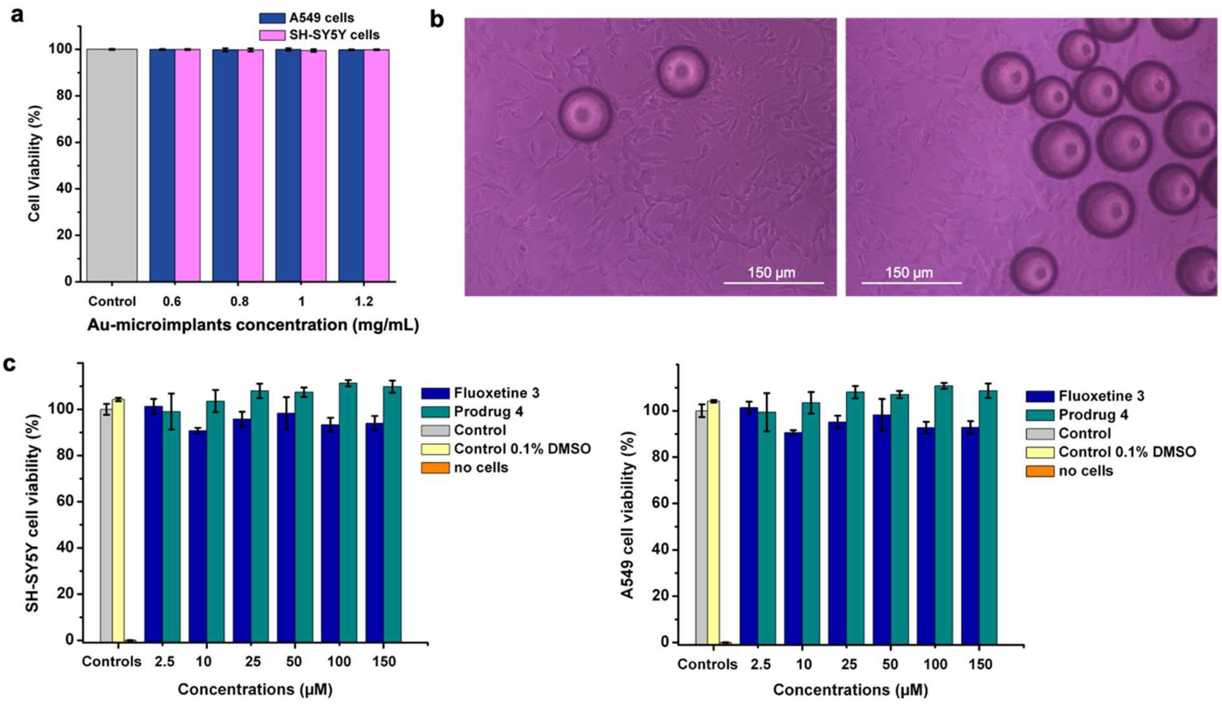

Figure S8. Cell Viability studies. a) Cell viability study of the biocompatibility of **Au-microimplants** in A549 cells and SH-SY5Y cells. **b)** Images from an optical microscope of SH-SY5Y cells with 1 mg/mL of **Au-microimplants** after 7 days of treatment. **Au-microimplants** can be identified as spheres of approx. 75 µm in average diameter. **c)** Cell viability study after 7 d treatment with 2.5, 10, 25, 50, 100 and 150 µM of Fluoxetine **3** and prodrug **4** in SH-SY5Y cells (left) and A549 cells (right). Error bars: ± SD from n = 3.

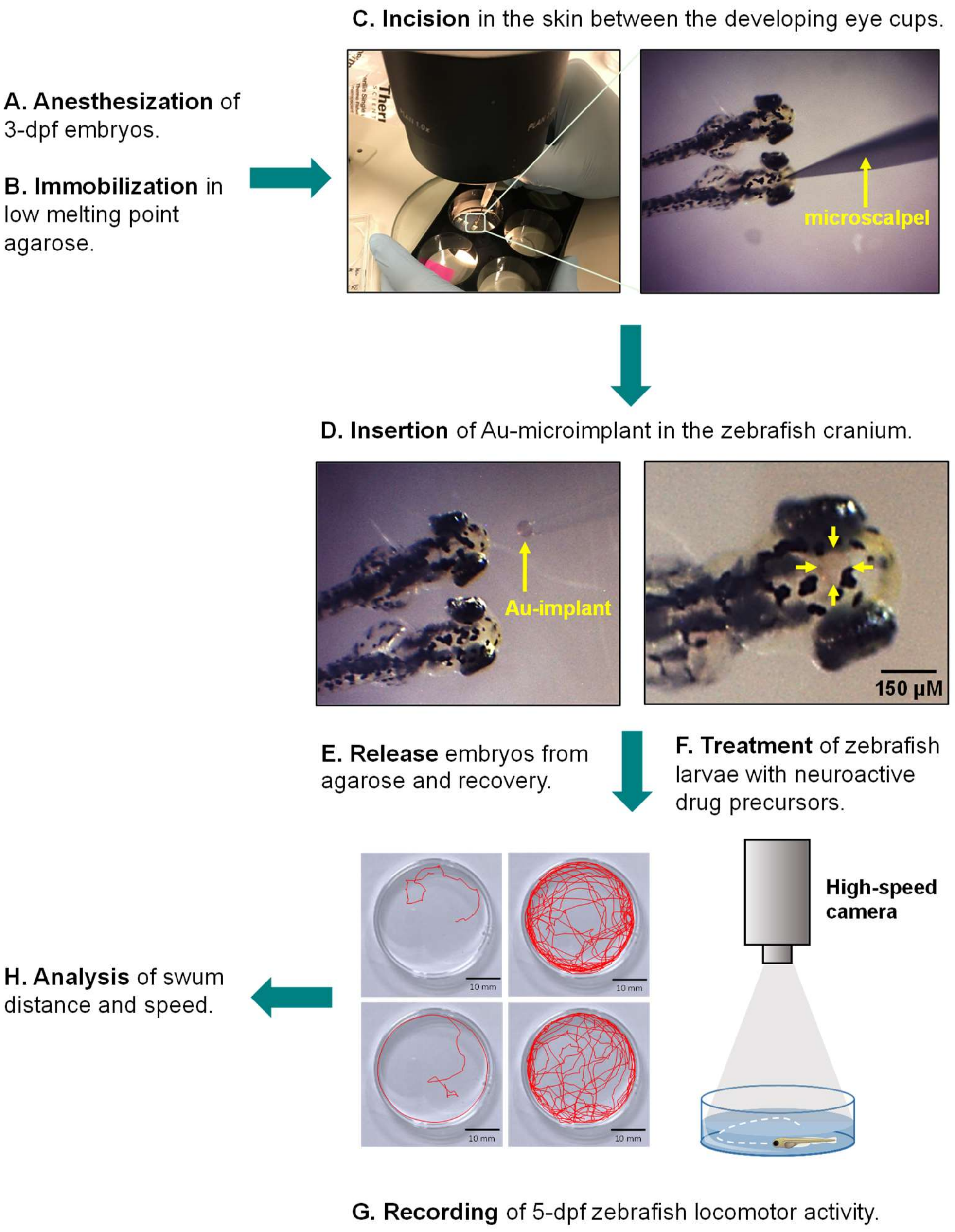

Figure S9. Overview of the implantation procedure in the cranium of 3-dpf zebrafish larvae, prodrug/DMSO treatment and screening of locomotor activity (steps A-H).

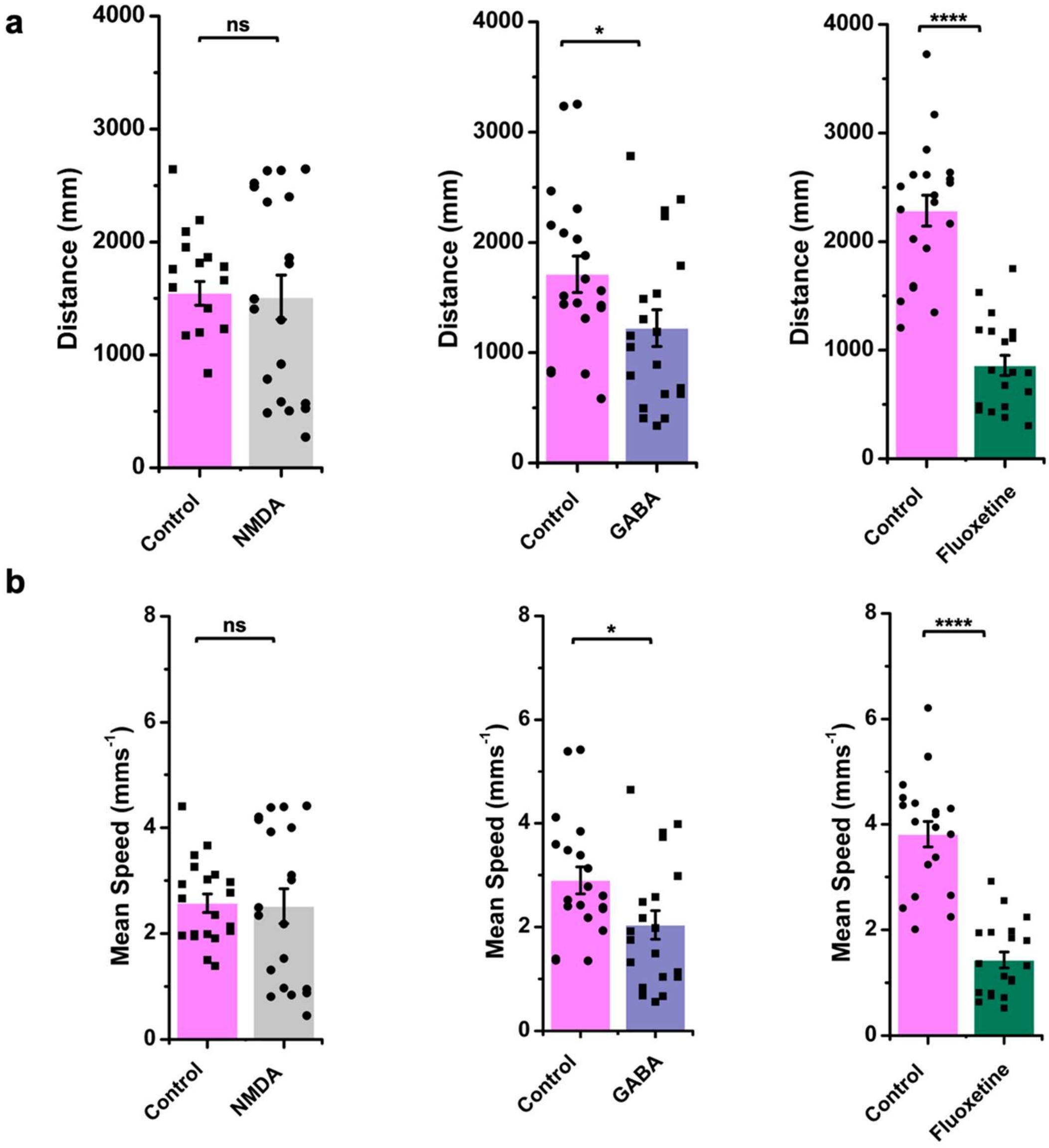

Figure S10. Influence of psychotropic drugs on locomotor activity in 5 dpf larval zebrafish. a) Distance travelled and **b)** speed by zebrafish in a 10-minute window following 2 h pre-incubation with NMDA (100 µM), GABA (150 µM) and fluoxetine (**1**, 50 µM). Error bars: ± SEM; n = 20.

RESULTS HIGHLIGHTS: The effects of psychotropic drugs on larval zebrafish behavior were varied. Incubation with NMDA had no effect on measured swimming parameters. 150 µM GABA produced a decrease in average speed (83.85% ± 16.35% of that measured with DMSO controls) and a small but insignificant decrease in distance (86.61% ± 16.31% of that measured with DMSO controls). 50 µM fluoxetine significantly reduced the mean total distance to 37.79% ± 3.22% and the mean swimming speed to 37.81% ± 3.21% of that measured with DMSO control values.

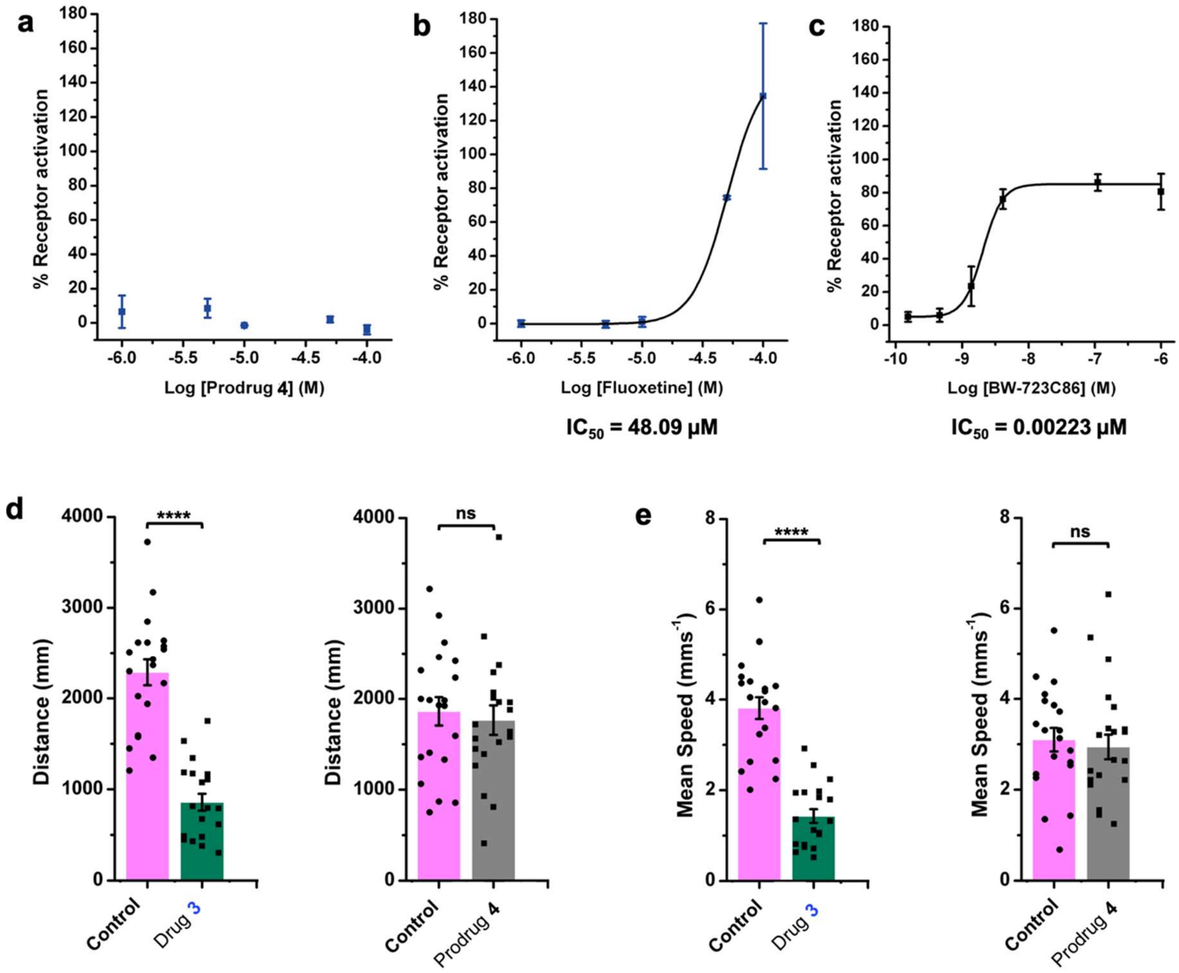

Figure S11. Analysis of the pharmacological properties of Fluoxetine 3 and prodrug 4. a-c) Agonist activity in 5-HT2B-expressing cells. a) Prodrug **4**, **b)** Fluoxetine **3**, and **c)** BW-723C86 (positive control). Dose response curve and IC_{50} value were generated by OriginPro 8 software. Error bars: ± SD from n = 2. **d,e) Effects of 3 and 4 on 5 dpf larval zebrafish swimming.** Larvae were treated with 50 μM of **3** or **4** in solution with 1% DMSO in zebrafish media for 2 h or 44 h, respectively. Control recordings were made in 1% DMSO in zebrafish media. **d)** Distance travelled and **e)** speed by zebrafish in a 10-minute window following 2 h pre-incubation with **3** or 44 h pre-incubation with prodrug **4**. Error bars: ± SEM; n = 20.

RESULTS HIGHLIGHTS: 50 μM drug **3** reduced mean total distance to 37.79% ± 3.22% of that measured with DMSO controls. Mean swim speed was 37.81% ± 3.21% lower of that measured with DMSO controls. Treatment with 50 μM prodrug **4** had no effect on these swimming parameters. These data indicate that drug **3** can modulate spontaneous larval swimming behaviour, and that its propargyl carbamate derivative **4** is effective in preventing its action *in vivo*.

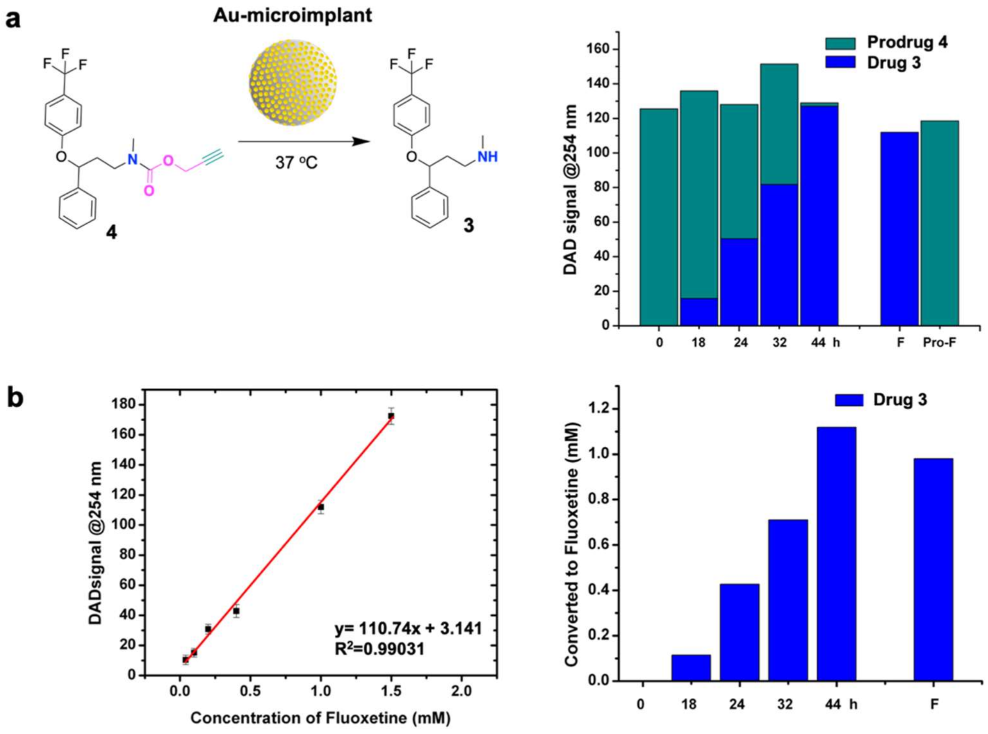

Figure S12. Prodrug-into-drug activation study using HPLC detection. a) Conversion of **4** (1.2 mM) into Fluoxetine **3**, mediated by **Au-microimplants** (1 mg/mL) in H_2O:MeOH 7:3 at 37°C obtained from HPLC/MSD measurements for DAD signal. **b)** Calibration curve of Fluoxetine **3** in H_2O:MeOH 7:3 (left) and reaction yields (right) obtained from HPLC/MSD measurements for DAD signal. Calibration equation is obtained by fitting a linear regression line to the collected data; R^2 is the coefficient of determination.

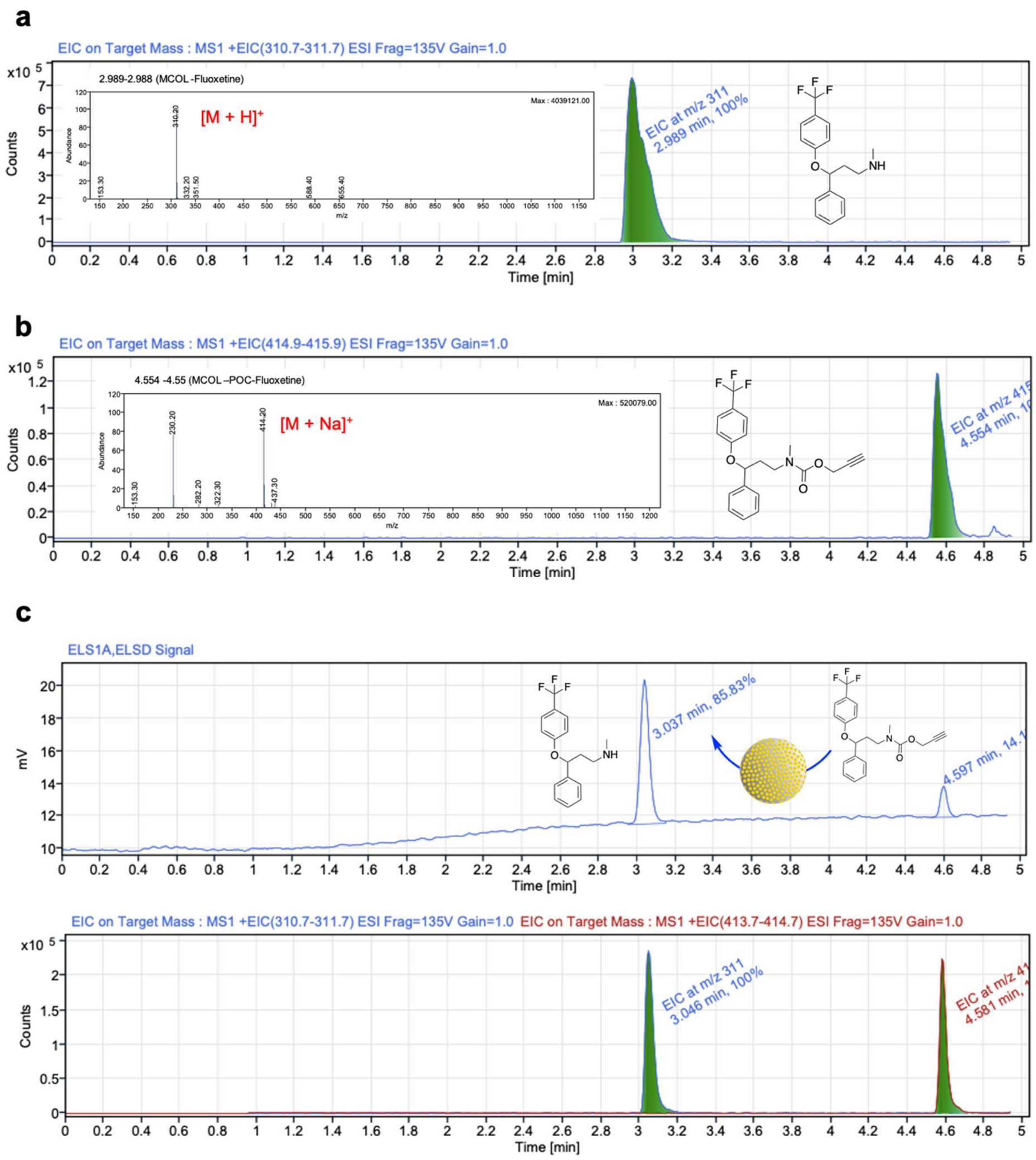

Figure S13. HPLC/MSD analysis of the reaction. HPLC-EIC chromatogram and ELSD-MS spectra (insert) of **a)** drug **3** and **b)** prodrug **4**. **c)** HPLC-ELSD (top) and -EIC chromatogram (bottom) of the reaction of **Au-microimplants** (1 mg/mL) and prodrug **4** (1.2 mM) after 44 h.

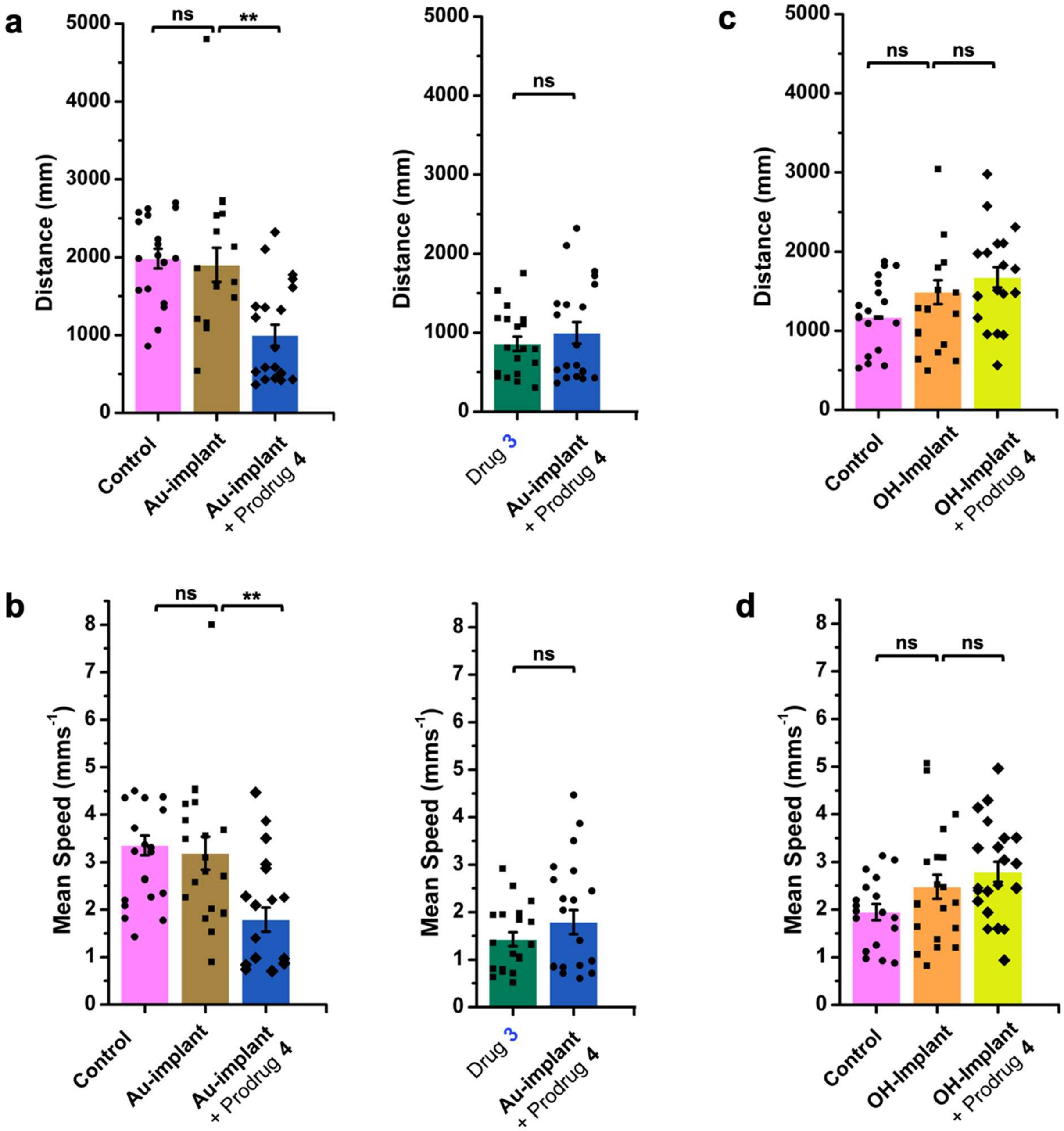

Figure S14. Comparative analysis of the modification of zebrafish swimming behaviour under different treatment conditions. Zebrafish larvae were implanted with a single implant (**Au-microimplant** or non-catalytic **OH-microimplant)** at 3dpf and incubated for 44 h in 1 % DMSO or in 50 μM of prodrug **4**. **a)** Distance travelled and **b)** swimming speed by zebrafish in a 10-minute window after treatment with DMSO (**control**), **Au-microimplant** only and **Au-microimplant** with prodrug **4**. Error bars: ± SEM; n = 22. **c,d)** Effects of **OH-microimplants** (catalytically inactive; negative control) on larval zebrafish swimming. **c)** Distance travelled and **d)** speed by zebrafish in a 10-minute window after treatment with DMSO (**control**), **OH-microimplant** only and **OH-microimplant** with prodrug **4**. Error bars: ± SEM; n = 20.

RESULTS HIGHLIGHTS: Au-implants alone had no effect on measured parameters compared to control (1% DMSO). 50 μM prodrug **4** treatment of zebrafish grafted with **Au-microimplants** reduced mean total distance to 50.34% ± 6.92% of that measured with **Au-microimplant** controls. Mean swim speed was 53.53% ± 7.52% of that measured with **Au-Implant** control values. Bioorthogonal intracranial release of **3** by **Au-microimplants** had similar effect to direct treatment with fluoxetine in modulating spontaneous zebrafish larval swimming. Neither mean total distance nor mean speed of larvae grafted with **OH-microimplants** was influenced by 44 h incubation with prodrug **4**.
